# Epidemiologic Trends in *Clostridioides difficile* Infections in a Regional Community Hospital Network

**DOI:** 10.1001/jamanetworkopen.2019.14149

**Published:** 2019-10-30

**Authors:** Nicholas A. Turner, Steven C. Grambow, Christopher W. Woods, Vance G. Fowler, Rebekah W. Moehring, Deverick J. Anderson, Sarah S. Lewis

**Affiliations:** 1 Division of Infectious Diseases, Duke University Medical Center, Durham, North Carolina; 2 Duke Center for Antimicrobial Stewardship and Infection Prevention, Durham, North Carolina; 3 Duke University, Department of Biostatistics and Bioinformatics, Durham, North Carolina; 4 Durham Veterans Affairs Health System, Durham, North Carolina

## Abstract

**Importance:**

*Clostridioides difficile* infection (CDI) remains a leading cause of health care facility–associated infection. A greater understanding of the regional epidemiologic profile of CDI could inform targeted prevention strategies.

**Objectives:**

To assess trends in incidence of health care facility–associated and community-acquired CDI among hospitalized patients over time and to conduct a subanalysis of trends in the NAP1 strain of CDI over time.

**Design, Setting, and Participants:**

This long-term multicenter cohort study reviewed records of patients (N = 2 025 678) admitted to a network of 43 regional community hospitals primarily in the southeastern United States from January 1, 2013, through December 31, 2017. Generalized linear mixed-effects models were used to adjust for potential clustering within facilities and changing test method (nucleic acid amplification testing or toxin enzyme immunoassay) over time.

**Main Outcomes and Measures:**

*Clostridioides difficile* infection incidence rates were counted as cases per 1000 admissions for community-acquired and total CDI cases or cases per 10 000 patient-days for health care facility–associated CDI. Long-term trends in the proportion of cases acquired in the community and in NAP1 strain incidence were also evaluated.

**Results:**

A total of 2 025 678 admissions and 21 254 CDI cases were included (12 678 [59.6%] female; median [interquartile range] age, 69 [55-80] years). Median (interquartile range) total CDI incidence increased slightly from 7.9 (3.5-12.4) cases per 1000 admissions in 2013 to 9.3 (4.9-13.7) cases per 1000 admissions in 2017. After adjustment, the overall incidence of health care facility–associated CDI declined (incidence rate ratio [IRR], 0.995; 95% CI, 0.990-0.999; *P* = .03), whereas insufficient evidence was found for either an increase or a decrease in community-acquired CDI (IRR, 1.004; 95% CI, 0.999-1.009; *P* = .14). The proportion of cases classified as community acquired increased over time from a mean (SD) of 0.49 (0.28) in 2013 to 0.61 (0.26) in 2017 (odds ratio, 1.010 per month; 95% CI, 1.006-1.015; *P* < .001). Rates of the NAP1 strain of CDI varied widely between facilities, with no statistically significant change in NAP1 strain incidence over time in the community setting (IRR, 1.007; 95% CI, 0.994-1.021) or health care facility setting (IRR, 1.011; 95% CI, 0.990-1.032).

**Conclusions and Relevance:**

The findings suggest that, despite the modest improvement in health care facility–associated CDI rates, a better understanding of community-acquired CDI incidence is needed for future infection prevention efforts.

## Key Points

**Question:**

What are the current trends in incidence rates of community-acquired and health care facility–associated *Clostridioides difficile* in US community hospitals?

**Findings:**

In this multicenter cohort study of 2 025 678 admissions and 21 254 cases of *Clostridioides difficile* infection, the incidence of health care facility–associated *C difficile* infection decreased from 2013 to 2017, whereas the incidence of community-acquired *C difficile* infection during the same period showed no statistically significant change. The proportion of cases classified as community acquired increased over time.

**Meaning:**

The findings suggest that the proportion of community-acquired *C difficile* infections is increasing over time and warrants further study to identify the factors behind this trend.

## Teaser

This cohort study examines the 5-year incidence of community-acquired and health care facility–associated *Clostridioides difficile* infection among patients in US community hospitals.

## Introduction

Approximately 500 000 cases of *Clostridioides difficile* infection (CDI) occur each year in the United States and are associated with more than 29 000 deaths.^1^ *Clostridioides difficile* infection is the leading cause of health care–associated diarrhea, is the most common health care–associated infection,^2^ and is an important health care–associated pathogen, but its epidemiologic profile is still evolving. In the early 2000s, the fluoroquinolone-resistant NAP1/027 strain of *C difficile* triggered multiple nosocomial epidemics, cementing CDI’s reputation as a predominantly health care facility–associated infection.^3,4,5^ Over the past decade, however, CDI has been increasingly reported among populations without previous hospital exposure.^6,7,8^ This type of infection is termed *community-acquired CDI*.

Robust population-based data on the relative incidence of health care facility–associated and community-acquired CDI are scarce because CDI is not a reportable disease, and testing might be performed through a variety of health care settings. In addition, the method of testing for CDI varies over time and between facilities, with the increasing adoption of the highly sensitive (although less specific) nucleic acid amplification testing (NAAT). Consequently, the true burden of community-acquired CDI and its risk factors remain poorly understood.

Despite the challenges in defining the true incidence of community-acquired and health care facility–associated CDI, future CDI prevention efforts depend on an accurate understanding of disease trends. An increase in community-acquired CDI burden would require a parallel shift in infection control practices, which are currently primarily centered in hospitals. Although all CDI definitions carry some risk of misclassification, the use of a single, consistent surveillance definition still permits a valid framework for assessing trends in incidence over time. We therefore undertook this multicenter cohort study to estimate the long-term trends in incidence of community-acquired and health care facility–associated CDI cases among a network of regional hospitals. Aside from using a robust data set spanning a range of community hospitals, this study is the first, to our knowledge, to adjust for changing test methods on such a large scale.

## Methods

### Data Collection

We conducted a retrospective, multicenter cohort study of admissions and CDI cases in the Duke Infection Control Outreach Network (DICON) from January 1, 2013, to December 31, 2017. Analysis was performed from April 2018 to August 2019. The DICON surveillance method has been previously described.^9^ The study design was reviewed and determined to be exempt by the institutional review board of Duke University Health System. Informed consent for data collection was waived because all relevant epidemiologic data were abstracted by each participating site without transfer of any protected health information. We followed the Strengthening the Reporting of Observational Studies in Epidemiology (STROBE) reporting guideline.

During the study period, DICON comprised 43 hospitals located in Georgia, Florida, North Carolina, South Carolina, Virginia, and West Virginia. *Clostridioides difficile* infections were defined according to the National Health Safety Network (NHSN) LabID definition: cases were identified by either a positive laboratory test result for *C difficile* from a toxin A/B assay or a positive laboratory test result for *C difficile* from the NAAT method using stool samples submitted to each hospital’s clinical microbiological laboratory.^10^ Any repeat positive result samples sent within 14 days were considered to be duplicate samples from the same episode and were not counted separately. By the same definition, community-acquired CDI was defined as a positive *C difficile* test result obtained on hospital day 1, 2, or 3 in patients who had not been admitted to the index facility in the previous 28 days. Health care facility–associated CDI merged the definitions of hospital-onset CDI (a positive test result obtained >3 days after admission and before discharge) with community-onset, health care facility–associated CDI (a positive test result obtained on hospital day 1, 2, or 3 from a patient who had been discharged from the index facility within the past 28 days).

Infection preventionists for each hospital prospectively collected data on the age, sex, race/ethnicity (as self-reported by the participants in their local medical record), source of admission (eg, home, skilled nursing facility, extended care facility, or another hospital), and results of the NAP1 strain test (if conducted) through reviewing paper medical records and a centralized, secure electronic database. For the 26 hospitals that reported incidence of the NAP1 strain, we also surveyed the prevalence of this epidemic strain over time. The NAP1 strain was identified by the commercial polymerase chain reaction–based assays in use at each site. Sites were excluded from analysis for any month in which they failed to report CDI cases and admissions data, in which case it would not be possible to calculate incidence.

The outcomes of CDI incidence rates were counted as cases per 1000 admissions for community-acquired and total CDI cases or cases per 10 000 patient-days (a standard measure in health care epidemiology representing the product of the number of patients at risk multiplied by the number of days at risk) for health care facility–associated CDI, in line with the Society of Healthcare Epidemiology for America (SHEA) reporting guidelines.^11^ We also evaluated long-term trends in NAP1 strain incidence, stratified by community-acquired and health care facility–associated cases. The analysis of NAP1 strain incidence was limited to those hospitals that tested for and reported the NAP1 strain.

### Statistical Analysis

We used generalized linear mixed-effects models with a log link for the long-term modeling of counts of CDI incidence. Four separate incidence rate models were constructed, one each for community-acquired CDI, health care facility–associated CDI, community-acquired NAP1 strain of CDI, and health care facility–associated NAP1 strain of CDI. Fixed effects in each model included hospital type (community vs academic), setting (urban vs rural), time (in months), and testing method (NAAT vs toxin enzyme immunoassay), modeled as a time-varying covariate. Each model also included random intercepts and slopes for time with an unstructured covariance matrix.

We hypothesized that the testing method was an important confounder of CDI incidence over time because the use of NAAT was associated with a higher sensitivity or lower specificity for CDI and was increasingly implemented over the period of interest.^12^ Monthly admissions were used as an offset term for community-acquired CDI and monthly patient-days as an offset term for health care facility–associated CDI. All models were constructed using the glmmTMB package in R, version 3.5.0 (R Project for Statistical Computing). All graphs were created using ggplot2.^13,14^

Data were assessed for evidence of zero inflation (the more frequent occurrence of zero-valued observations) and overdispersion. As CDI incidence rates exhibited evidence of overdispersion, a negative binomial response distribution was assumed. Inspection of quantile-to-quantile plots suggested the negative binomial distribution was a reasonable assumption. Adjustment for zero inflation did not improve model fit and did not markedly alter the effect estimates. Fitted rather than actual plots were constructed as a final visual inspection of each model’s accuracy (complete R code with model specification and plots used to test assumptions are presented in the eAppendix in the Supplement).

We constructed a separate mixed-effects logistic regression model for long-term modeling of the proportion of community-acquired CDI cases compared with total CDI cases with the same fixed-effects structure as the count models described earlier. The model was constructed using the glmmTMB package and included random intercepts and slopes for time with an unstructured covariance matrix. Adjustments for hospital type, setting, and testing method were included.

Throughout this study, a statistical significance threshold of 2-sided *P* = .05 was used. *P* values for effect estimates in each model were calculated by Wald χ^2^ test.

## Results

**Figure 1.**
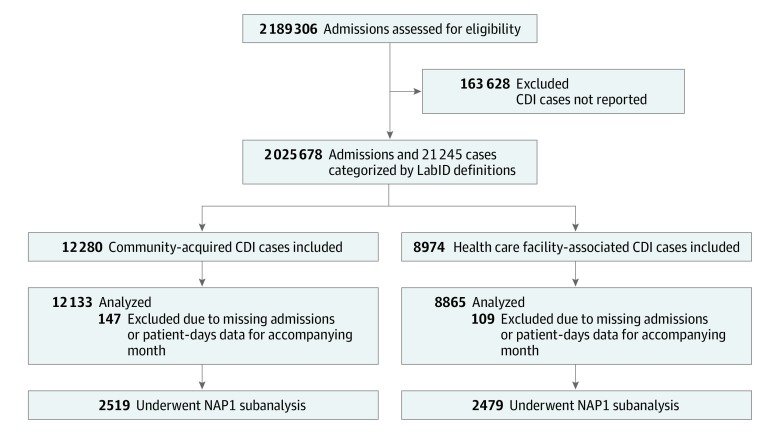
Flow Diagram of *Clostridioides difficile* Infection (CDI) Cases

The cohort included 2 189 306 admissions to 43 hospitals in DICON from January 1, 2013, to December 31, 2017. We excluded admissions for which accompanying CDI counts were not available (n = 163 628) or instances in which CDI cases were reported without accompanying admissions data (n = 256 CDI cases). A total of 2 025 678 admissions and 21 254 CDI cases were analyzed (Figure 1). Most months with either missing CDI case counts or missing admissions data came from 3 smaller centers with mean admissions per month of fewer than 100; 1 of the excluded centers was an ambulatory surgery center with admissions limited to brief postprocedure observational stays. Basic features of the included hospitals are listed in Table 1.

**Table 1.** Characteristics of Included Hospitals

Variable	No. (%)
Total included, No.	43
Licensed beds, median (IQR), No.	210 (141-303)
Monthly admissions, median (IQR), No.	775 (422-1242)
Geographic location	
Florida	2 (5)
Georgia	9 (21)
North Carolina	23 (54)
South Carolina	3 (7)
Virginia	5 (12)
West Virginia	1 (2)
Setting	
Urban	29 (67)
Rural	14 (33)
Type	
Teaching or academic	18 (42)
Community	25 (58)
*Clostridioides difficile* test method	
Toxin enzyme immunoassay only	5 (12)
Transitioned from toxin enzyme immunoassay to NAAT	13 (30)
NAAT only	25 (58)
Hospitals reporting NAP1 strain incidence	26 (61)

Abbreviations: IQR, interquartile range; NAAT, nucleic acid amplification testing.

**Figure 2.**
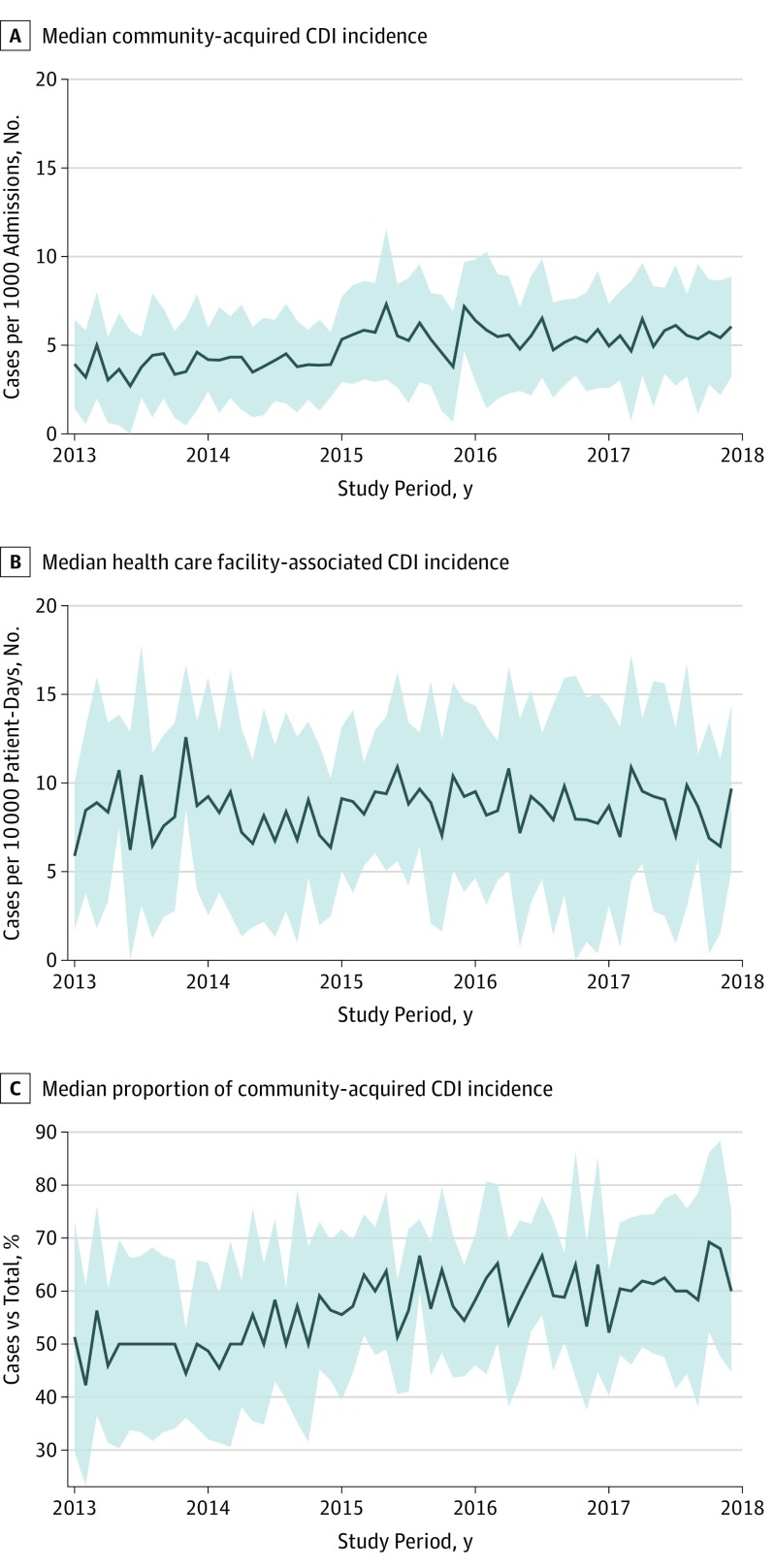
Observed Trends in *Clostridioides difficile* Infection (CDI) Incidence by Year in 43 Included Hospitals Solid line indicates unadjusted median; gray shaded area, interquartile range.

Median (interquartile range [IQR]) total CDI incidence increased slightly from 7.9 (3.5-12.4) cases per 1000 admissions in 2013 to 9.3 (4.9-13.7) cases per 1000 admissions in 2017 (eFigure 1 in the Supplement). Median (IQR) community-acquired CDI incidence increased from 3.7 (0.9-6.6) cases per 1000 admissions in 2013 to 5.6 (2.6-8.6) cases per 1000 admissions in 2017 (Figure 2). Both community-acquired and total CDI incidence rates showed a possible inflection point occurring in 2015. On further investigation, the apparent inflection point corresponded temporally with 9 hospitals transitioning to polymerase chain reaction–based testing. Over the same period, median (IQR) health care facility–associated CDI incidence appeared stable, ranging from 8.4 (2.8-14.1) cases per 10 000 patient-days in 2013 to 8.5 (2.9-14.1) cases per 10 000 patient-days in 2017 (Figure 2). Based on a survey of the subset of hospitals reporting the NAP1 strain (n = 26), the proportion of cases attributable to the NAP1 strain was 22.6%. The proportion of CDI cases attributable to the NAP1 strain increased from 22.5% in 2013 to 24.6% in 2017; however, this proportion varied widely between facilities.

Among CDI cases, the median (IQR) age was 69 (55-80) years, and 12 678 patients (59.6%) were female. Several differences were observed between patients with community-acquired CDI and those with health care facility–associated CDI. Patients with health care facility–associated CDI were, in general, older (median [IQR], 70 [58-80] years vs 67 [53-79] years) and more often male than patients with community-acquired CDI (eTable 1 in the Supplement). Although health care facility–associated CDI cases had health care contact by definition, a substantial proportion of community-acquired CDI cases still had recent previous contact with a facility that was not captured by the LabID surveillance definition. For example, contact with a skilled nursing facility or dialysis center would not be counted as health care facility–associated CDI. Admission source was unknown or unreported for a substantial number of community-acquired cases. Consequently, 1502 community-acquired CDI cases (12.2%) were recently admitted to another health care facility; 3097 (25.2%) had no admission to the index facility and lacked documentation of previous outside health care contact.

Table 2 summarizes mixed-effects modeling measurement estimates for community-acquired CDI, health care facility–associated CDI, community-acquired NAP1 strain of CDI, and health care facility–associated NAP1 strain of CDI. Molecular testing was associated with incidence rate ratios (IRRs) of 1.919 (95% CI, 1.653-2.228; *P* < .001) for community-acquired CDI and 1.831 (95% CI, 1.591-2.108; *P* < .001) for health care facility–associated CDI. Time, measured in months, was modeled as both a random effect and a fixed effect and served as the primary covariate of interest. Adjustment for molecular testing resulted in lower IRRs for time than in the unadjusted models, supporting the need for adjustment to avoid inflation of CDI incidence rates over time with the increasing adoption of NAAT methods.

**Table 2.** Model-Based Measurement Estimates

Variable	Adjustment	Fixed Effects	IRR or OR (95% CI)^a^	*P* Value
Incidence models				
Community-acquired CDI	Unadjusted	Time, mo	1.007 (1.001-1.013)	.01
	Adjusted	Time, mo	1.004 (0.999-1.009)	.14
		Setting, urban relative to rural	0.862 (0.623-1.193)	.37
		Type, academic vs community	0.999 (0.738-1.352)	.99
		Test type (NAAT vs antigen)	1.919 (1.653-2.228)	<.001
Health care facility–associated CDI	Unadjusted	Time, mo	0.998 (0.993-1.004)	.49
	Adjusted	Time, mo	0.995 (0.990-0.999)	.03
		Setting, urban vs rural	0.802 (0.631-1.020)	.07
		Type, academic vs community	1.026 (0.823-1.278)	.82
		Test type, NAAT vs antigen	1.831 (1.591-2.108)	<.001
Community-acquired NAP1 strain of CDI	Unadjusted	Time, mo	1.007 (0.994-1.021)	.31
Health care facility–associated NAP1 strain of CDI	Unadjusted	Time, mo	1.011 (0.990-1.032)	.32
Proportion model				
Community-acquired CDI as proportion of total CDI cases	Unadjusted	Time, mo	1.010 (1.006-1.015)	<.001
	Adjusted	Time, mo	1.010 (1.006-1.014)	<.001
		Setting, urban vs rural	1.056 (0.815-1.369)	.68
		Type, academic vs community	0.847 (0.674-1.065)	.16
		Test type, NAAT vs antigen	1.083 (0.904-1.296)	.39

Abbreviations: CDI, *Clostridioides difficile* infection; IRR, incidence rate ratio; NAAT, nucleic acid amplification testing; OR, odds ratio.

^a^ Values are IRRs for incidence models and OR for proportion model.

**Figure 3.**
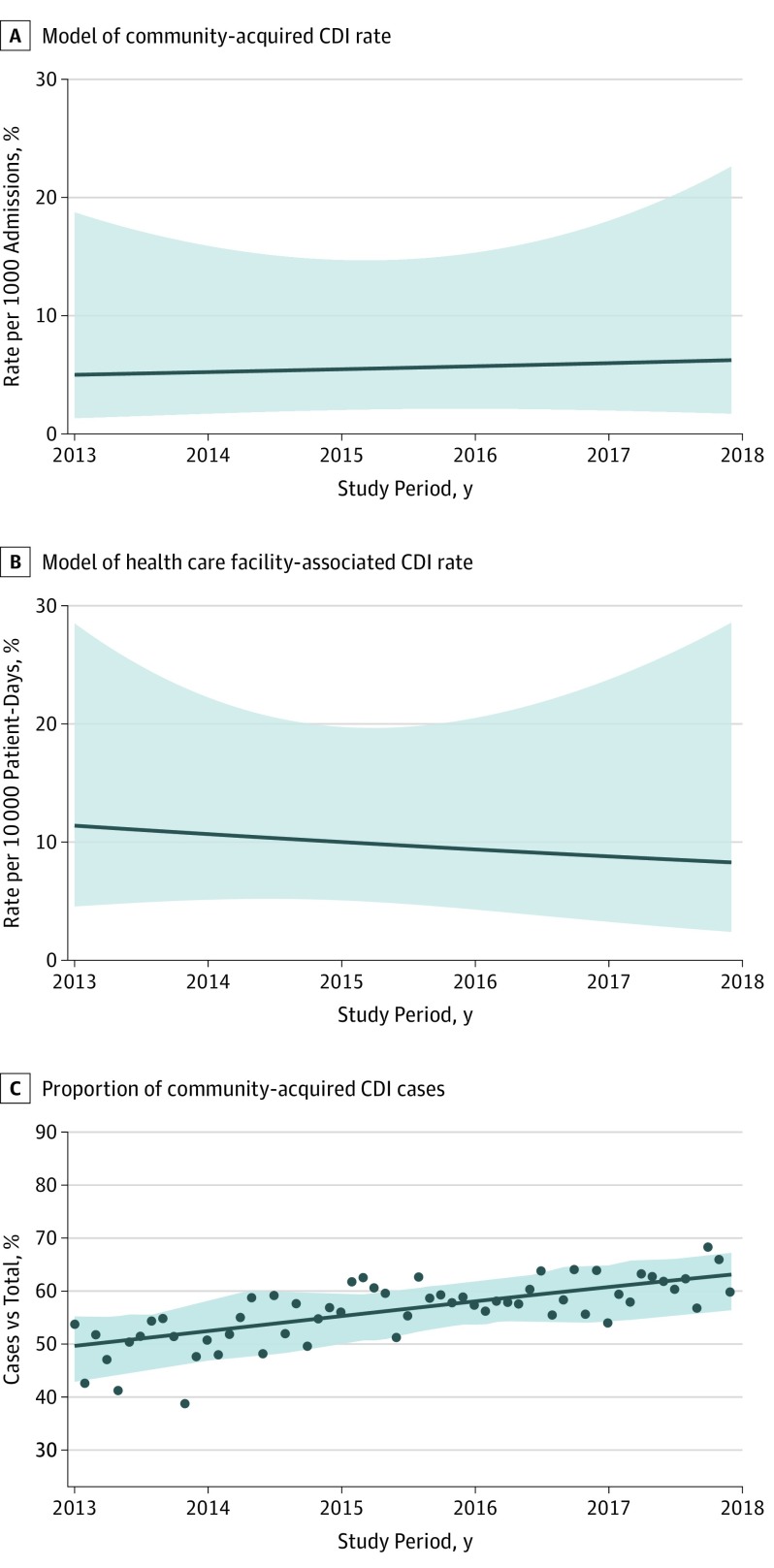
Estimated Trends in *Clostridioides difficile* Infection (CDI) Incidence Rates Over Time for a Hospital A and B, Shaded area represents 95% CI for the model, reflecting the degree of variation between health care facilities. Estimated community-acquired CDI incidence is reported for an urban community hospital with a mean of 941 admissions per month using nucleic acid amplification testing (NAAT) CDI testing. Estimated health care facility–associated CDI incidence is reported for an urban community hospital with a mean of 4078 patient-days per month using NAAT CDI testing. C, Scatterplot points represent mean proportion of community-acquired CDI cases compared with total CDI cases across all facilities for each month in the study period. The line indicates modeled proportion of community-acquired CDI cases using mixed-effects logistic regression; shaded area, 95% CI for the model.

When accounting for test type, the incidence of health care facility–associated CDI decreased by a mean of 0.5% per month (monthly IRR, 0.995; 95% CI, 0.990-0.999; *P* = .03); community-acquired CDI incidence increased by a mean of 0.4% per month (monthly IRR, 1.004; 95% CI, 0.999-1.009; *P* = .14). Figure 3 displays estimated incidence rates for an urban community hospital in DICON (mean size, 941 admissions per month) using NAAT. Although the community-acquired CDI model showed a modestly positive slope, it did not reach statistical significance. In contrast, health care facility–associated CDI declined modestly over time, and the decreasing incidence remained statistically significant. To further investigate the test method over time, we conducted post hoc modeling of community-acquired and health care facility–associated CDI incidence rates, stratified by whether facilities changed their test method during the study period. Consistent with the modeled results, facilities that used the same test method throughout the study period showed no evidence of either an increase or a decrease in community-acquired CDI incidence over time (IRR, 1.001; 95% CI, 0.995-1.007; *P* = .79). However, a decrease in health care facility–associated CDI incidence was found (IRR, 0.993; 95% CI, 0.986-0.999; *P* = .02) (eTable 2 in the Supplement).

On a separate analysis of the proportion of community-acquired CDI cases compared with the total CDI case burden within DICON, the proportion of community-acquired CDI cases increased from a median (IQR) of 48.5% (31.9%-65.2%) in 2013 to 61.0% (47.1%-74.9%) in 2017 (Figure 2). Logistic regression was used to model the change in proportion of community-acquired CDI cases over time, with an increase in odds ratio (OR) of community-acquired CDI by 1% per month from a mean (SD) of 0.49 (0.28) in 2013 to 0.61 (0.26) in 2017 (OR, 1.010; 95% CI, 1.006-1.015; *P* < .001) (Table 2 and Figure 3).

The NAP1 strain trends were limited to the 26 hospitals with the capability of testing for NAP1 strain by polymerase chain reaction. Although cases with the NAP1 strain were a minority in all CDI cases in the region, the trends were complex. After constructing long-term models for community-acquired and health care facility–associated NAP1 strain of CDI over time, we found substantial variation in NAP1 strain rates by hospital. Some facilities had NAP1 strain rates more than 10-fold higher than their peers (eFigure 2 in the Supplement). A close inspection of these trends over time suggests that these outlier facilities likely experienced outbreaks driven by the NAP1 strain given that discrete increases in NAP1 strain case rates were seen over relatively short periods (eg, 3 to 6 months). Partly owing to the substantial variation in NAP1 strain incidence rates between facilities, adjusted NAP1 strain incidence rates exhibited large CIs. The NAP1 strain showed no statistically significant increase over time in either community-acquired CDI (IRR, 1.007; 95% CI, 0.994-1.021; *P* = .31) or health care facility–associated CDI (IRR, 1.011; 95% CI, 0.990-1.032; *P* = .32) (Table 2).

## Discussion

The primary goal of this study was to assess recent trends in community-acquired and health care facility–associated CDI incidence rates. In particular, we sought to reassess the trends since the sharp increase in community-acquired CDI cases was first noted in the early 2000s.^15,16^ This large-scale, long-term analysis demonstrated that, although health care facility–associated CDI rates decreased modestly over time, community-acquired CDI rates did not. Consequently, community-acquired CDI composes an increasing proportion of the CDI burden encountered by US hospitals. The reasons for the higher proportion of CDI cases in the community remain unclear but could signal the existence of independent reservoirs of CDI in the community. We believe the greater number of cases warrants further investigation into the phenomenon of community-acquired CDI. Improving the design of CDI prevention efforts requires knowledge of the risk factors and sources of community-acquired CDI.

Several features of this study aid the assessment of CDI incidence rates over time. The study population was large, including more than 2 million admissions into 43 hospitals. All study sites used the same NHSN case definition throughout the study period. Mixed-effects modeling accounted for the correlation between repeated measurements and potential case clustering within sites. Furthermore, because the study database included information on the test methods used over time at each site, we were able to account for the rate inflation associated with sites transitioning from toxin enzyme immunoassay to NAAT.

Although CDI incidence rates are reported in a variety of ways, the incidence rates in this study generally agreed with those in published research that used laboratory-based definitions and the same denominators.^17,18^ One of the largest recent surveys on health care facility–associated infections found no substantial change in health care facility–associated CDI rates across 10 hospitals in the Centers for Disease Control and Prevention Emerging Infections Program from 2011 to 2015.^8^ Because data on test methods were not available, the authors of the previous study noted the possibility that increasing use of NAAT might have masked an actual decrease in CDI rates over time.^8^ By incorporating data on NAAT use at each of the survey sites for the present study, we were able to measure and account for the implications of changing test methods over time. Use of molecular testing was associated with increased CDI incidence rates. The IRRs of 1.8 to 1.9 with molecular testing were generally consistent with previous estimates.^19^ After accounting for the increased use of NAAT over time, health care facility–associated CDI rates showed a modest but statistically significant decrease over time.

Although it was somewhat encouraging that health care facility–associated CDI rates were modestly decreasing, the same trend was not seen in total CDI or community-acquired CDI rates. As a result, the proportion of CDI cases classified as community-acquired CDI increased, affirming the ongoing relevance of community-acquired CDI since their high rates were first noted in the early 2000s.^15,16^ The reason for this trend is unclear, and there are multiple factors that will require further investigation to better define. Surveillance definitions do not capture all potential health care–related exposures, and so many CDI cases may be misattributed to the community setting. To address this issue, we conducted a post hoc sensitivity analysis with more stringent definitions for health care facility–associated CDI, including all patients admitted from a skilled nursing facility, extended care facility, or another hospital. Under the more stringent case definition, community-acquired CDI still showed a nonsignificant increase over time. Health care facility–associated CDI was still estimated to have decreased over time, but it was no longer statistically significant (eTable 3 in the Supplement). Although the overall trends remained the same, our analysis indicated that trends in community-acquired CDI compared with health care facility–associated CDI were sensitive to the definitions used. Future research into community-acquired CDI will need to be mindful of the additional health care exposures not incorporated into the LabID definition.

Among hospitals that reported NAP1 strain test results, NAP1 strain represented a minority among the total CDI cases within the region, a finding similar to the 22.0% reported in a recent survey of Veterans Health Administration hospitals.^20^ Consistent with its first description as a hypervirulent pathogen prone to epidemics, NAP1 strain’s behavior in the region was defined by periodic outbreaks. Subanalysis of NAP1 strain trends was limited to much smaller sample sizes, but in general, the NAP1 strain remained relatively constrained in time and geographic scope without clear evidence of any substantial increase in incidence over time.

### Limitations

This study has several limitations. First, tracking community-acquired CDI incidence is particularly difficult. Because CDI is not a reportable illness and testing might be performed through a variety of health care settings, complete case detection would require either a central laboratory or fully integrated health care system. Consequently, our analysis was limited to assessment of CDI among patients who were admitted to the hospital within a set 5-year period. Although DICON represents a diverse group of hospitals primarily in the southeastern United States, the network does not include all inpatient facilities within the catchment area and does not include any outpatient facilities. Even the assessment of health care facility–associated CDI incidence has its limitations. Second, current NHSN LabID surveillance methods are based solely on microbiological results. Consequently, NHSN case definitions cannot distinguish colonized from infected patients and are highly affected by testing practices. Estimates of asymptomatic carriage range from 5% to 15% among patients presenting to health care facilities, making the identification of infected compared with colonized patients problematic.^21,22,23,24^

Third, methods of testing for CDI vary over time and between facilities. The NAAT method is more sensitive than the traditional toxin enzyme immunoassay testing. Consequently, greater use of NAAT over time confounds the long-term assessment of CDI incidence.^19,25,26^ Fourth, even the categorization of community-acquired CDI and health care facility–associated CDI varies by the definition used. For ease of reporting, facility-centered surveillance definitions consider health care contact only at the index facility. If a wider range of health care contacts are considered, up to 23% of community-acquired CDI cases might be reclassified as health care facility–associated CDI.^17^ Fifth, NAP1 strain rates were based on voluntary reporting, with the potential for selection bias in which facilities chose to report. Sixth, the observed increase in the proportion of community-acquired CDI cases might be, at least in part, associated with changing testing practices. For example, many hospitals have launched initiatives to promote early recognition of diarrhea and testing for CDI to reduce the misclassification of community-acquired CDI cases as hospital-onset cases, which are publicly reported and associated with financial penalties for hospitals. A post hoc analysis of the change in time from admission to testing over the study period did reveal a trend toward earlier testing over time (eFigure 3 in the Supplement). Without data on time of symptom onset and appropriateness of testing, however, it is impossible to identify whether this change is from an actual increase in community-acquired CDI incidence or from unmeasured changes in testing practices.

## Conclusions

This study provides large-scale, robust evidence of a modest reduction in health care facility–associated CDI incidence but largely unchanged or even slightly increased community-acquired CDI rates. If this trend continues beyond the study period, focusing future research efforts on improving the definitions for and surveillance of community-acquired CDI will be essential. Without an understanding of the sources, risk factors, and transmission of CDI within the community, efforts to improve health care facility–associated CDI incidence alone are unlikely to curtail overall CDI rates.

## Notes

Supplement. **eAppendix.** Complete R Code With Model Specification and Plots **eFigure 1.** Total CDI Incidence by Year Among 43 DICON Hospitals **eFigure 2.** Variation in NAP1 Incidence Across Hospitals **eFigure 3.** Trend in Time From Admission to CDI Testing Over the Study Period **eTable 1.** Demographic and Clinical Features of CA- and HCFA-CDI Cases **eTable 2.** Results of Modeling CA- and HCFA-CDI Incidence Rates Over Time, Stratified by Whether Test Method Changed Over Time **eTable 3.** Results of Sensitivity Analysis
