# Supplementary material for: Epidemiologic Trends in Clostridioides difficile Infections in a Regional Community Hospital Network
Source: JAMA Netw Open. 2019 Oct 30;2(10):e1914149. doi: 10.1001/jamanetworkopen.2019.14149 (PMC6824221; doi:10.1001/jamanetworkopen.2019.14149)
Supplement: Supplement. — eAppendix. Complete R Code With Model Specification and Plots eFigure 1. Total CDI Incidence by Year Among 43 DICON Hospitals eFigure 2. Variation in NAP1 Incidence Across Hospitals eFigure 3. Trend in Time From Admission to CDI Testing Over the Study Period eTable 1. Demographic and Clinical Features of CA- and HCFA-CDI Cases eTable 2. Results of Modeling CA- and HCFA-CDI Incidence Rates Over Time, Stratified by Whether Test Method Changed Over Time eTable 3. Results of Sensitivity Analysis [file jamanetwopen-2-e1914149-s001.pdf]

# Supplementary Online Content

Turner NA, Grambow SC, Woods CW, et al. Epidemiologic trends in *Clostridioides difficile* infections in a regional community hospital network. *JAMA Netw Open*. 2019;2(10):e1914149. doi:10.1001/jamanetworkopen.2019.14149

**eAppendix.** Complete R Code With Model Specification and Plots

**eFigure 1.** Total CDI Incidence by Year Among 43 DICON Hospitals

**eFigure 2.** Variation in NAP1 Incidence Across Hospitals

**eFigure 3.** Trend in Time From Admission to CDI Testing Over the Study Period

**eTable 1.** Demographic and Clinical Features of CA- and HCFA-CDI Cases

**eTable 2.** Results of Modeling CA- and HCFA-CDI Incidence Rates Over Time, Stratified by Whether Test Method Changed Over Time

**eTable 3.** Results of Sensitivity Analysis

This supplementary material has been provided by the authors to give readers additional information about their work.

© 2019 Turner NA et al. *JAMA Network Open*

**eAppendix.** Complete R Code With Model Specification and Plots

# DICON_C_diff_Modeling_Trials_Suppl_File_with_Extra_Ana

nat7

2019-09-09

```
### Final DICON C difficile Epidemiology Modeling Trials ###

# Supplemental R Code Files
# Version 9/4/19

# Modeling Script:
library(readr)

# Upload final cohorts:
ca_cdi_final <- read_csv("P:/DICON_OUTREACH/Research/Nick Turner ARLG Research/DICON CDI Basic Epi/ca_cdi_final.csv")
```

```
## Parsed with column specification:
## cols(
##   HospitalID = col_double(),
##   Year = col_double(),
##   Month = col_double(),
##   CA_CDI_cases = col_double(),
##   Molecular = col_double(),
##   HospitalIDLabel = col_character(),
##   CDiffTotalAdmissions = col_double(),
##   CDiffPatientDays = col_double(),
##   CDiffLabIDHO = col_double(),
##   CDiffPCR = col_double(),
##   Beds = col_double(),
##   Urban = col_double(),
##   Academic = col_character(),
##   incidence = col_double(),
##   incidence_a = col_double()
## )
```

```
hcfa_cdi_final <- read_csv("P:/DICON_OUTREACH/Research/Nick Turner ARLG Research/DICON CDI Basic Epi/hcfa_cdi_final.csv")
```

```
## Parsed with column specification:
## cols(
##   HospitalID = col_double(),
##   Year = col_double(),
##   Month = col_double(),
##   HCFA_CDI_cases = col_double(),
##   Molecular = col_double(),
##   HospitalIDLabel = col_character(),
##   CDiffTotalAdmissions = col_double(),
##   CDiffPatientDays = col_double(),
##   CDiffLabIDHO = col_double(),
##   CDiffPCR = col_double(),
##   Beds = col_double(),
##   Urban = col_double(),
##   Academic = col_character(),
##   incidence = col_double(),
##   incidence_a = col_double()
## )
```

```
# Import Hospital Data:
DICON_Hospital_Data <- read_csv("P:/DICON_OUTREACH/Research/Nick Turner ARLG Research/DICON CDI Basic Epi/DICON_Hospital_Dat
a.csv")
```

```
## Parsed with column specification:
## cols(
##   HospitalID = col_double(),
##   HospitalIDLabel = col_character(),
##   Beds = col_double(),
##   Urban = col_double(),
##   Academic = col_character(),
##   County = col_character(),
##   State = col_character(),
##   USDA_hog = col_double(),
##   USDA_cattle = col_double(),
##   USDA_poultry = col_double()
## )
```

```
# Count cases from Marquette, MI:
library(tidyverse)
```

```
## -- Attaching packages --------------------------------------------------------------- tidyverse 1.2.1 --
```

```
## v ggplot2 3.2.0     v purrr   0.3.2
## v tibble  2.1.3     v dplyr   0.8.2
## v tidyr   0.8.3     v stringr 1.4.0
## v ggplot2 3.2.0     v forcats 0.4.0
```

```
## -- Conflicts ------------------------------------------------------------------ tidyverse_conflicts() --
## x dplyr::filter() masks stats::filter()
## x dplyr::lag()    masks stats::lag()
```

```
ca_mi <- ca_cdi_final %>% filter(ca_cdi_final$HospitalID==1053)
hcfa_mi <- hcfa_cdi_final %>% filter(hcfa_cdi_final$HospitalID==1053)

sum(ca_mi$CA_CDI_cases)
```

```
## [1] 17
```

```
sum(ca_mi$CDiffTotalAdmissions)
```

```
## [1] 10373
```

```
sum(hcfa_mi$HCFA_CDI_cases)
```

```
## [1] 28
```

```
sum(hcfa_mi$CDiffPatientDays)
```

```
## [1] 43300
```

```
# Exclude Marquette MI (site 1053) from analysis:
ca_cdi_final <- ca_cdi_final %>% filter(ca_cdi_final$HospitalID!=1053)
hcfa_cdi_final <- hcfa_cdi_final %>% filter(hcfa_cdi_final$HospitalID!=1053)

# Create a combined date category to allow plotting by month:
library(zoo)
```

```
##
## Attaching package: 'zoo'
```

```
## The following objects are masked from 'package:base':
##
##     as.Date, as.Date.numeric
```

© 2019 Turner NA et al. *JAMA Network Open*

```
ca_cdi_final$date <- as.yearmon(paste(ca_cdi_final$Year, ca_cdi_final$Month),"%Y %m")
hcfa_cdi_final$date <- as.yearmon(paste(hcfa_cdi_final$Year, hcfa_cdi_final$Month),"%Y %m")

# Make new date column as factor:
ca_cdi_final[["date2"]] <- as.factor(ca_cdi_final[["date"]])
hcfa_cdi_final[["date2"]] <- as.factor(hcfa_cdi_final[["date"]])

# Make a new date column as numeric, by month:
ca_cdi_final[["date3"]] <- as.numeric(ca_cdi_final[["date2"]])
hcfa_cdi_final[["date3"]] <- as.numeric(hcfa_cdi_final[["date2"]])

# Assure HospitalID, year, month are all character values:
ca_cdi_final$HospitalID <- as.character(ca_cdi_final$HospitalID)
ca_cdi_final$Year <- as.character(ca_cdi_final$Year)
ca_cdi_final$Month <- as.character(ca_cdi_final$Month)

hcfa_cdi_final$HospitalID <- as.character(hcfa_cdi_final$HospitalID)
hcfa_cdi_final$Year <- as.character(hcfa_cdi_final$Year)
hcfa_cdi_final$Month <- as.character(hcfa_cdi_final$Month)

# Add HospitalID as a factor (required for glmmADMB modeling):
ca_cdi_final[["HospitalID2"]] <- as.factor(ca_cdi_final[["HospitalID"]])
hcfa_cdi_final[["HospitalID2"]] <- as.factor(hcfa_cdi_final[["HospitalID"]])

# Evaluate general trends:
# Spaghetti plots:
interaction.plot(x.factor = ca_cdi_final[["date2"]], trace.factor=ca_cdi_final[["HospitalIDLabel"]], response=ca_cdi_final
[["incidence_a"]], type="l", legend=FALSE, col=1:60, main="CA-CDI Incidence (per 1,000 admissions)", xlab="Month", ylab="Inc
idence per 1,000 admits")
axis(side = 1, at=1:60, labels=FALSE)
```

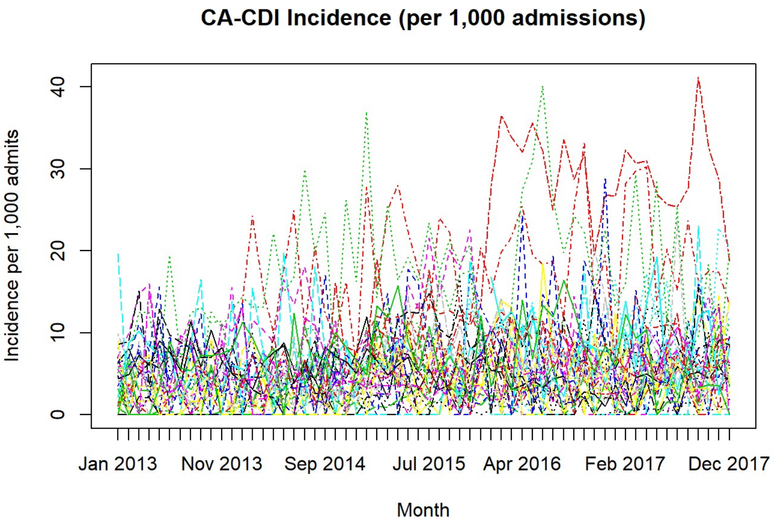


```
interaction.plot(x.factor = hcfa_cdi_final[["date2"]], trace.factor=hcfa_cdi_final[["HospitalIDLabel"]], response=hcfa_cdi_f
inal[["incidence"]], type="l", legend=FALSE, col=1:60, main="HCFA-CDI Incidence (per 10,000 patient-days)", xlab="Month", yl
ab="Incidence per 10,000 patient-days")
axis(side = 1, at=1:60, labels=FALSE)
```

© 2019 Turner NA et al. *JAMA Network Open*

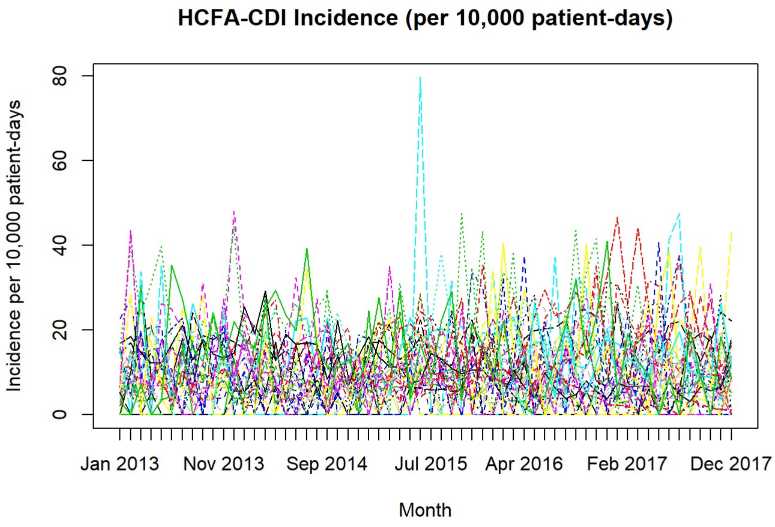


```
### Phase I Data Exploration ###

## Ia: Examine for Potential Outliers
# By Boxplot:
boxplot(ca_cdi_final$CA_CDI_cases)
```

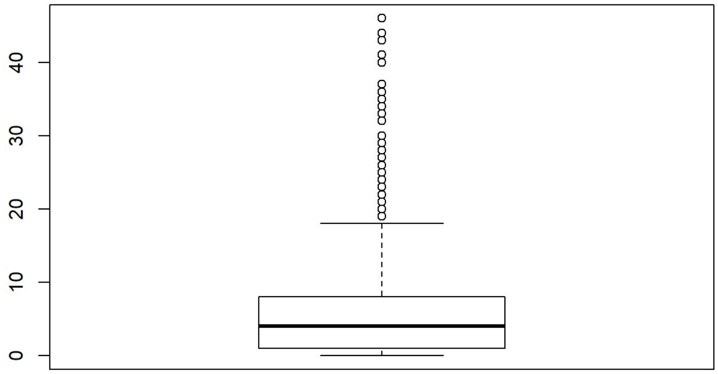


```
boxplot(hcfa_cdi_final$HCFA_CDI_cases)
```

© 2019 Turner NA et al. *JAMA Network Open*

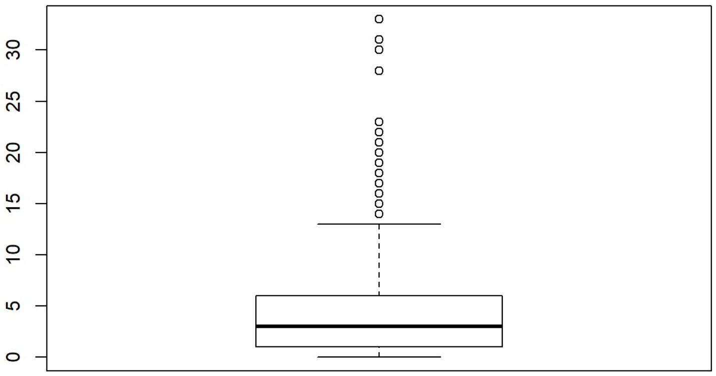


```
# By Cleveland plot:
dotchart(ca_cdi_final$CA_CDI_cases, xlab="Cases per month", ylab="Order of data")
```

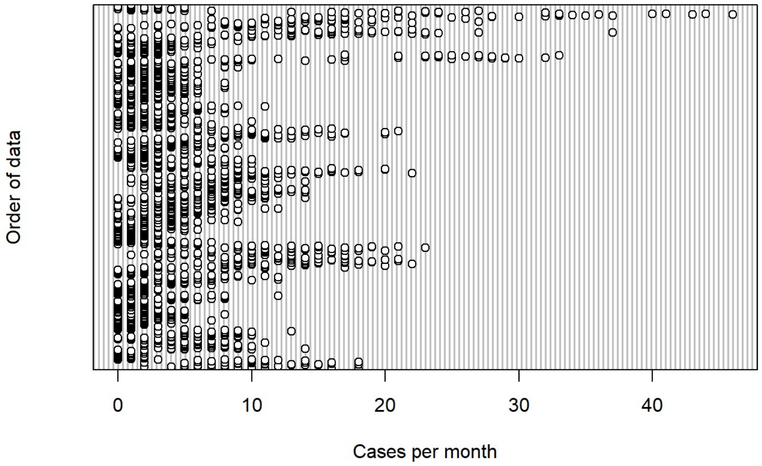


```
dotchart(hcfa_cdi_final$HCFA_CDI_cases, xlab="Cases per month", ylab="order of data")
```

© 2019 Turner NA et al. *JAMA Network Open*

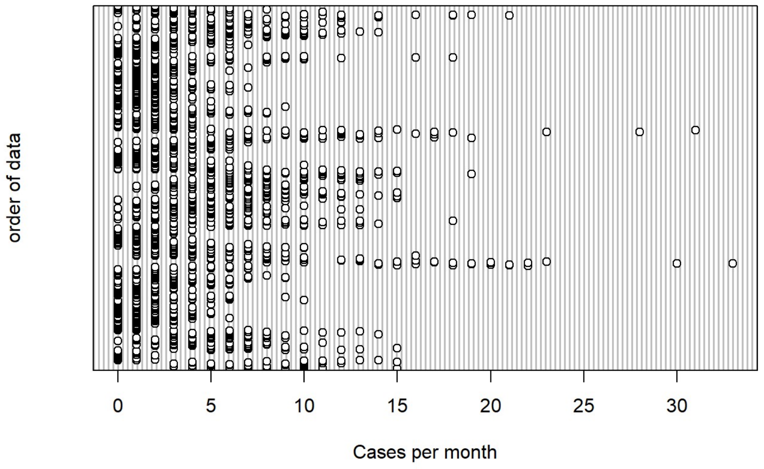


```
dotchart(hcfa_cdi_final$CDiffTotalAdmissions, xlab="Admissions per month", ylab="order of data")
```

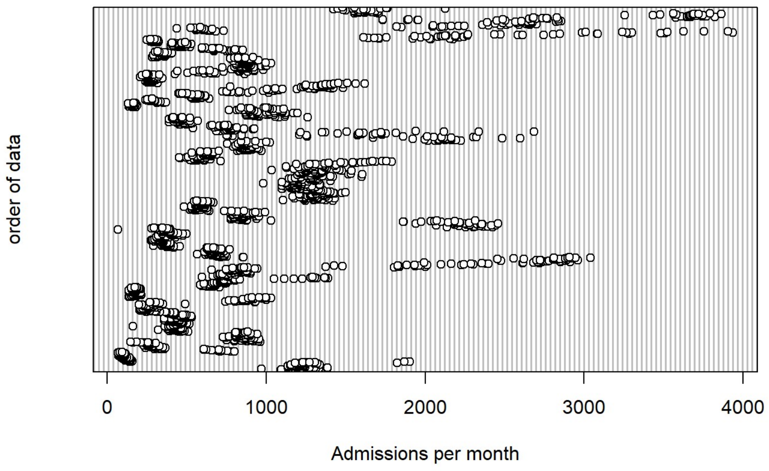


```
# General inspection by Lattice Plots
library(lattice)

# CA Plots
xtabs(~ HospitalID + date3, ca_cdi_final)
```

© 2019 Turner NA et al. *JAMA Network Open*

```
##           date3
## HospitalID 1 2 3 4 5 6 7 8 9 10 11 12 13 14 15 16 17 18 19 20 21 22 23 24
##       1001 1 1 1 1 1 1 1 1 1  1  1  1  1  1  1  1  1  1  1  1  1  1  1  1
##       1002 1 1 1 1 1 1 1 1 1  1  1  1  1  1  1  1  1  1  1  1  1  1  1  1
##       1003 1 1 1 1 1 1 1 1 1  1  1  1  1  1  1  1  1  1  1  1  1  1  1  1
##       1004 1 1 1 1 1 1 1 1 1  1  1  1  1  1  1  1  1  1  1  1  1  1  1  1
##       1005 1 1 1 1 1 1 1 1 1  1  1  1  1  1  1  1  1  1  1  1  1  1  1  1
##       1006 1 1 1 1 1 1 1 1 1  1  1  1  1  1  1  1  1  1  1  1  1  1  1  1
##       1008 1 1 1 1 1 1 1 1 1  1  1  1  1  1  1  1  1  1  1  1  1  1  1  1
##       1009 1 1 1 1 1 1 1 1 1  1  1  1  1  1  1  1  1  1  1  1  1  1  1  1
##       1010 1 1 1 1 1 1 1 1 1  1  1  1  1  1  1  1  1  1  1  1  1  1  1  1
##       1011 1 1 1 1 1 1 1 1 1  1  1  1  1  1  1  1  1  1  1  1  1  1  1  1
##       1012 1 1 1 1 1 1 1 1 1  1  1  1  1  1  1  1  1  1  1  1  1  1  1  1
##       1017 1 1 1 1 1 1 1 1 1  1  1  1  1  1  1  1  1  1  1  1  1  1  1  1
##       1019 1 1 1 1 1 1 1 1 1  1  1  1  1  1  1  1  1  1  1  1  1  1  1  1
##       1021 1 1 1 1 1 1 1 1 1  1  1  1  1  1  1  1  1  1  1  1  1  1  1  1
##       1022 1 1 1 1 1 1 1 1 1  1  1  1  1  1  1  1  1  1  1  1  1  1  1  1
##       1023 1 1 1 1 1 1 1 1 1  1  1  1  1  1  1  1  1  1  1  1  1  1  1  1
##       1024 1 1 1 1 1 1 1 1 1  1  1  1  1  1  1  1  1  1  1  1  1  1  1  1
##       1026 1 1 1 1 1 1 1 1 1  1  1  1  1  1  1  1  1  1  1  1  1  1  1  1
##       1028 1 1 1 1 1 1 1 1 1  1  1  1  1  1  1  1  1  1  1  1  1  1  1  1
##       1029 1 1 1 1 1 1 1 1 1  1  1  1  1  1  1  1  1  1  1  1  1  1  1  1
##       1031 1 1 1 1 1 1 1 1 1  1  1  1  1  1  1  1  1  1  1  1  1  1  1  1
##       1033 1 1 1 1 1 1 1 1 1  1  1  1  1  1  1  1  1  1  1  1  1  1  1  1
##       1036 1 1 1 1 1 1 1 1 1  1  1  1  1  1  1  1  1  1  1  1  1  1  1  1
##       1039 1 1 1 1 1 1 1 1 1  1  1  1  1  1  1  1  1  1  1  1  1  1  1  1
##       1040 1 1 1 1 1 1 1 1 1  1  1  1  1  1  1  1  1  1  1  1  1  1  1  1
##       1043 1 1 1 1 1 1 1 1 1  1  1  1  1  1  1  1  1  1  1  1  1  1  1  1
##       1044 1 1 1 1 1 1 1 1 1  1  1  1  1  1  1  1  1  1  1  1  1  1  1  1
##       1045 1 1 1 1 1 1 1 1 1  1  1  1  1  1  1  1  1  1  1  1  1  1  1  1
##       1046 1 1 1 1 1 1 1 1 1  1  1  1  1  1  1  1  1  1  1  1  1  1  1  1
##       1047 1 1 1 1 1 1 1 1 1  1  1  1  1  1  1  1  1  1  1  1  1  1  1  1
##       1048 1 1 1 1 1 1 1 1 1  1  1  1  1  1  1  1  1  1  1  1  1  1  1  1
##       1049 1 1 1 1 1 1 1 1 1  1  1  1  1  1  1  1  1  1  1  1  1  1  1  1
##       1050 1 1 1 1 1 1 1 1 1  1  1  1  1  1  1  1  1  1  1  1  1  1  1  1
##       1051 0 0 0 0 0 0 0 0 0  0  0  0  0  0  0  0  0  0  1  1  1  1  1  0
##       1052 0 0 0 0 0 0 0 0 0  0  0  0  0  0  0  0  0  0  0  0  0  0  0  0
##       1054 0 0 0 0 0 0 0 0 0  0  0  0  0  0  0  0  0  0  0  0  0  0  0  0
##       1055 0 0 0 0 0 0 0 0 0  0  0  0  0  0  0  0  0  0  0  0  0  0  0  0
##       1056 0 0 0 0 0 0 0 0 0  0  0  0  0  0  0  0  0  0  0  0  0  0  0  0
##       1057 0 0 0 0 0 0 0 0 0  0  0  0  1  1  1  1  1  1  1  1  1  1  1  1
##       1058 0 0 0 0 0 0 0 0 0  0  0  0  0  0  0  0  0  0  0  0  0  0  0  0
##       1060 0 0 0 0 0 0 0 0 0  0  0  0  1  1  1  1  1  1  1  1  1  1  1  1
##       1062 0 0 0 0 0 0 0 0 0  0  0  0  0  0  0  0  0  0  0  0  0  0  0  0
##       1063 0 0 0 0 0 0 0 0 0  0  0  0  0  0  0  0  0  0  0  0  0  0  0  0
##           date3
## HospitalID 25 26 27 28 29 30 31 32 33 34 35 36 37 38 39 40 41 42 43 44 45
##       1001  1  1  1  1  1  1  1  1  1  1  1  1  1  1  1  1  1  1  1  1  1
##       1002  1  1  1  1  1  1  1  1  1  1  1  1  1  1  1  1  1  1  1  1  1
##       1003  1  1  1  1  1  1  1  1  1  1  1  1  1  1  1  1  1  1  1  1  1
##       1004  1  1  1  1  1  1  1  1  1  1  1  1  1  1  1  1  1  1  1  1  1
##       1005  1  1  1  1  1  1  1  1  1  1  1  1  1  1  1  1  1  1  1  1  1
##       1006  1  1  1  1  1  1  1  1  1  1  1  1  1  1  1  1  1  1  1  1  1
##       1008  1  1  1  1  1  1  1  1  1  1  1  1  1  1  1  1  1  1  1  1  1
##       1009  1  1  1  1  1  1  0  0  0  0  0  0  0  0  0  0  0  0  0  0  0
##       1010  1  1  1  1  1  1  1  1  1  1  1  1  1  1  1  1  1  1  1  1  1
##       1011  1  1  1  1  1  1  1  1  1  1  1  1  1  1  1  1  1  1  1  1  1
##       1012  1  1  1  1  1  1  1  1  1  1  1  1  1  1  1  1  1  1  1  1  1
##       1017  1  1  1  1  1  1  1  1  1  1  1  1  1  1  1  1  1  1  1  1  1
##       1019  1  1  1  1  1  1  1  1  1  1  1  1  1  1  1  1  1  1  1  1  1
##       1021  1  1  1  1  1  1  1  1  1  1  1  1  1  1  1  1  1  1  1  1  1
##       1022  1  1  1  1  1  1  1  1  1  1  1  1  1  1  1  1  1  1  1  1  1
##       1023  1  1  1  1  1  1  1  1  1  1  1  1  1  1  1  1  1  1  0  0  0
##       1024  1  1  1  1  1  1  1  1  1  1  1  1  1  1  1  1  1  1  1  1  1
##       1026  1  1  1  1  1  1  1  1  1  1  1  1  1  1  1  1  1  1  1  1  1
##       1028  1  1  1  1  1  1  1  1  1  1  1  1  1  1  1  1  1  1  1  1  1
##       1029  1  1  1  1  1  1  1  1  1  1  1  1  1  1  1  1  1  1  1  1  1
##       1031  1  1  1  1  1  1  1  1  1  1  1  1  1  1  1  1  1  1  1  1  1
##       1033  1  1  1  1  1  1  1  1  1  1  1  1  1  1  1  1  1  1  1  1  1
##       1036  1  1  1  1  1  1  1  1  1  1  1  1  1  1  1  1  1  1  1  1  1
##       1039  1  1  1  1  1  1  1  1  1  1  1  1  1  1  1  1  1  1  1  1  1
##       1040  1  1  1  1  1  1  1  1  1  1  1  1  1  1  1  1  1  1  1  1  1
##       1043  1  1  1  1  1  1  1  1  1  1  1  1  1  1  1  1  1  1  1  1  1
##       1044  0  0  0  0  0  0  0  0  0  0  0  0  0  0  0  0  0  0  0  0  0
##       1045  1  1  1  1  1  1  1  1  1  1  1  1  1  1  1  1  1  1  1  1  1
##       1046  1  1  1  0  0  0  0  0  0  0  0  0  0  0  0  0  0  0  0  0  0
##       1047  1  1  1  1  1  1  1  1  1  1  1  1  1  1  1  1  1  1  1  1  1
##       1048  1  1  1  1  1  1  1  1  1  1  1  1  1  1  1  1  1  1  1  1  1
##       1049  1  1  1  1  1  1  1  1  1  1  1  1  1  1  1  1  1  1  1  1  1
##       1050  1  1  1  1  1  1  1  1  1  1  1  1  1  1  1  1  1  1  1  1  1
##       1051  1  1  1  1  1  1  1  1  1  1  1  1  1  1  1  1  1  1  1  1  1
```

© 2019 Turner NA et al. *JAMA Network Open*

```
##       1052  1  1  1  1  1  1  1  1  1  1  1  1  1  1  1  1  1  1  1  1  1
##       1054  0  0  0  0  0  0  1  1  1  1  1  1  1  1  1  1  1  1  1  1  1
##       1055  0  0  0  0  0  0  0  0  0  0  0  0  1  1  1  1  1  1  1  1  1
##       1056  0  0  0  0  0  0  0  0  0  0  0  0  1  1  1  1  1  1  1  1  1
##       1057  1  1  1  1  1  1  1  1  1  1  1  1  1  1  1  1  1  1  1  1  1
##       1058  0  0  0  0  0  0  0  0  0  0  0  0  1  1  1  1  1  1  1  1  1
##       1060  1  1  1  1  1  1  1  1  1  1  1  1  1  1  1  1  1  1  1  1  1
##       1062  1  1  1  1  1  1  1  1  1  1  1  1  1  1  1  1  1  1  1  1  1
##       1063  1  1  1  1  1  1  1  1  1  1  1  1  1  1  1  1  1  1  1  1  1
##           date3
## HospitalID 46 47 48 49 50 51 52 53 54 55 56 57 58 59 60
##       1001  1  1  1  1  1  1  1  1  1  1  1  1  1  1  1
##       1002  1  1  1  1  1  1  1  1  1  1  1  1  1  1  1
##       1003  1  1  1  1  1  1  1  1  1  1  1  1  1  1  1
##       1004  1  1  1  1  1  1  1  1  1  1  1  1  1  1  1
##       1005  1  1  1  1  1  1  1  1  1  1  1  1  1  1  1
##       1006  1  1  1  1  1  1  1  1  1  1  1  1  1  1  1
##       1008  1  1  1  1  1  1  1  1  1  1  1  1  1  1  1
##       1009  0  0  0  0  0  0  0  0  0  0  0  0  0  0  0
##       1010  1  1  1  1  1  1  1  1  1  1  1  1  1  1  1
##       1011  1  1  1  1  1  1  1  1  1  1  1  1  1  1  1
##       1012  1  1  1  1  1  1  1  1  1  1  1  1  1  1  1
##       1017  1  1  1  1  1  1  1  1  1  1  1  1  1  1  1
##       1019  1  1  1  1  1  1  1  1  1  1  1  1  1  1  1
##       1021  1  1  1  1  1  1  1  1  1  1  1  1  1  1  1
##       1022  1  1  1  1  1  1  1  1  1  1  1  1  1  1  1
##       1023  0  0  0  0  0  0  0  0  0  0  0  0  0  0  0
##       1024  1  1  1  1  1  1  1  1  1  1  1  1  1  1  1
##       1026  1  1  1  1  1  1  1  1  1  1  1  1  1  1  1
##       1028  1  1  1  1  1  1  1  1  1  1  1  1  1  1  1
##       1029  1  1  1  1  1  1  1  1  1  1  1  1  1  1  1
##       1031  1  1  1  1  1  1  1  1  1  1  1  1  1  1  1
##       1033  1  1  1  1  1  1  1  1  1  1  1  1  1  1  1
##       1036  1  1  1  1  1  1  1  1  1  1  1  1  1  1  1
##       1039  1  1  1  1  1  1  1  1  1  1  1  1  1  1  1
##       1040  1  1  1  1  1  1  1  1  1  1  1  1  1  1  1
##       1043  1  1  1  1  1  1  1  1  1  1  1  1  1  1  1
##       1044  0  0  0  0  0  0  0  0  0  0  0  0  0  0  0
##       1045  1  1  1  1  1  1  1  1  1  1  1  1  1  1  1
##       1046  0  0  0  0  0  0  0  0  0  0  0  0  0  0  0
##       1047  1  1  1  1  1  1  1  1  1  1  1  1  1  1  1
##       1048  1  1  1  1  1  1  1  1  1  1  1  1  1  1  1
##       1049  1  1  1  1  1  1  1  1  1  1  0  0  0  0  0
##       1050  1  1  1  1  1  1  1  1  1  1  1  1  1  1  1
##       1051  1  1  1  1  1  1  1  1  1  1  1  1  1  1  1
##       1052  1  1  1  1  1  1  1  1  1  1  1  1  1  1  1
##       1054  1  1  1  1  1  1  1  1  1  1  1  1  1  1  1
##       1055  1  1  1  1  1  1  1  1  1  1  1  1  1  1  1
##       1056  1  1  1  1  1  1  1  1  1  1  0  0  0  0  0
##       1057  1  1  1  1  1  1  1  1  1  1  1  1  1  1  1
##       1058  1  1  1  1  1  1  1  1  1  1  1  1  1  1  1
##       1060  1  1  1  1  1  1  1  1  1  1  1  1  1  1  1
##       1062  1  1  1  1  1  1  1  1  1  1  1  1  1  1  1
##       1063  1  1  1  1  1  1  1  1  1  1  1  1  1  1  1
```

```
print(xyplot(CA_CDI_cases ~ date3 | HospitalID, ca_cdi_final, aspect = "xy",
             layout = c(5,5), type = c("g", "p", "r"),
             index.cond = function(x,y) coef(lm(y ~ x))[1],
             xlab = "Month",
             ylab = "CA cases"))
```

© 2019 Turner NA et al. *JAMA Network Open*

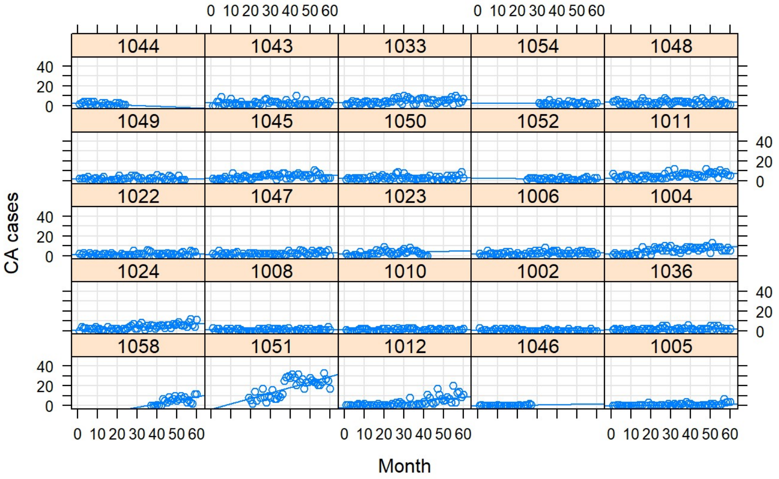


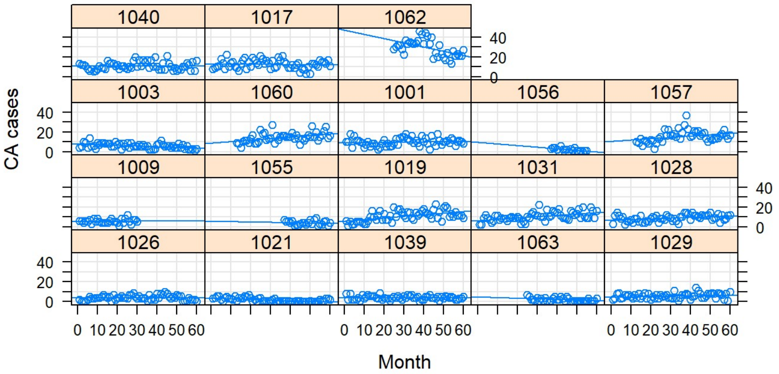


```
# HCFA Plots
xtabs(~ HospitalID + date3, hcfa_cdi_final)
```

© 2019 Turner NA et al. *JAMA Network Open*

```
##            date3
## HospitalID 1 2 3 4 5 6 7 8 9 10 11 12 13 14 15 16 17 18 19 20 21 22 23 24
##       1001 1 1 1 1 1 1 1 1 1  1  1  1  1  1  1  1  1  1  1  1  1  1  1  1
##       1002 1 1 1 1 1 1 1 1 1  1  1  1  1  1  1  1  1  1  1  1  1  1  1  1
##       1003 1 1 1 1 1 1 1 1 1  1  1  1  1  1  1  1  1  1  1  1  1  1  1  1
##       1004 1 1 1 1 1 1 1 1 1  1  1  1  1  1  1  1  1  1  1  1  1  1  1  1
##       1005 1 1 1 1 1 1 1 1 1  1  1  1  1  1  1  1  1  1  1  1  1  1  1  1
##       1006 1 1 1 1 1 1 1 1 1  1  1  1  1  1  1  1  1  1  1  1  1  1  1  1
##       1008 1 1 1 1 1 1 1 1 1  1  1  1  1  1  1  1  1  1  1  1  1  1  1  1
##       1009 1 1 1 1 1 1 1 1 1  1  1  1  1  1  1  1  1  1  1  1  1  1  1  1
##       1010 1 1 1 1 1 1 1 1 1  1  1  1  1  1  1  1  1  1  1  1  1  1  1  1
##       1011 1 1 1 1 1 1 1 1 1  1  1  1  1  1  1  1  1  1  1  1  1  1  1  1
##       1012 1 1 1 1 1 1 1 1 1  1  1  1  1  1  1  1  1  1  1  1  1  1  1  1
##       1017 1 1 1 1 1 1 1 1 1  1  1  1  1  1  1  1  1  1  1  1  1  1  1  1
##       1019 1 1 1 1 1 1 1 1 1  1  1  1  1  1  1  1  1  1  1  1  1  1  1  1
##       1021 1 1 1 1 1 1 1 1 1  1  1  1  1  1  1  1  1  1  1  1  1  1  1  1
##       1022 1 1 1 1 1 1 1 1 1  1  1  1  1  1  1  1  1  1  1  1  1  1  1  1
##       1023 1 1 1 1 1 1 1 1 1  1  1  1  1  1  1  1  1  1  1  1  1  1  1  1
##       1024 1 1 1 1 1 1 1 1 1  1  1  1  1  1  1  1  1  1  1  1  1  1  1  1
##       1026 1 1 1 1 1 1 1 1 1  1  1  1  1  1  1  1  1  1  1  1  1  1  1  1
##       1028 1 1 1 1 1 1 1 1 1  1  1  1  1  1  1  1  1  1  1  1  1  1  1  1
##       1029 1 1 1 1 1 1 1 1 1  1  1  1  1  1  1  1  1  1  1  1  1  1  1  1
##       1031 1 1 1 1 1 1 1 1 1  1  1  1  1  1  1  1  1  1  1  1  1  1  1  1
##       1033 1 1 1 1 1 1 1 1 1  1  1  1  1  1  1  1  1  1  1  1  1  1  1  1
##       1036 1 1 1 1 1 1 1 1 1  1  1  1  1  1  1  1  1  1  1  1  1  1  1  1
##       1039 1 1 1 1 1 1 1 1 1  1  1  1  1  1  1  1  1  1  1  1  1  1  1  1
##       1040 1 1 1 1 1 1 1 1 1  1  1  1  1  1  1  1  1  1  1  1  1  1  1  1
##       1043 1 1 1 1 1 1 1 1 1  1  1  1  1  1  1  1  1  1  1  1  1  1  1  1
##       1044 1 1 1 1 1 1 1 1 1  1  1  1  1  1  1  1  1  1  1  1  1  1  1  1
##       1045 1 1 1 1 1 1 1 1 1  1  1  1  1  1  1  1  1  1  1  1  1  1  1  1
##       1046 1 1 1 1 1 1 1 1 1  1  1  1  1  1  1  1  1  1  1  1  1  1  1  1
##       1047 1 1 1 1 1 1 1 1 1  1  1  1  1  1  1  1  1  1  1  1  1  1  1  1
##       1048 1 1 1 1 1 1 1 1 1  1  1  1  1  1  1  1  1  1  1  1  1  1  1  1
##       1049 1 1 1 1 1 1 1 1 1  1  1  1  1  1  1  1  1  1  1  1  1  1  1  1
##       1050 1 1 1 1 1 1 1 1 1  1  1  1  1  1  1  1  1  1  1  1  1  1  1  1
##       1051 0 0 0 0 0 0 0 0 0  0  0  0  0  0  0  0  0  0  1  1  1  1  1  0
##       1052 0 0 0 0 0 0 0 0 0  0  0  0  0  0  0  0  0  0  0  0  0  0  0  0
##       1054 0 0 0 0 0 0 0 0 0  0  0  0  0  0  0  0  0  0  0  0  0  0  0  0
##       1055 0 0 0 0 0 0 0 0 0  0  0  0  0  0  0  0  0  0  0  0  0  0  0  0
##       1056 0 0 0 0 0 0 0 0 0  0  0  0  0  0  0  0  0  0  0  0  0  0  0  0
##       1057 0 0 0 0 0 0 0 0 0  0  0  0  1  1  1  1  1  1  1  1  1  1  1  1
##       1058 0 0 0 0 0 0 0 0 0  0  0  0  0  0  0  0  0  0  0  0  0  0  0  0
##       1060 0 0 0 0 0 0 0 0 0  0  0  0  1  1  1  1  1  1  1  1  1  1  1  1
##       1062 0 0 0 0 0 0 0 0 0  0  0  0  0  0  0  0  0  0  0  0  0  0  0  0
##       1063 0 0 0 0 0 0 0 0 0  0  0  0  0  0  0  0  0  0  0  0  0  0  0  0
##            date3
## HospitalID 25 26 27 28 29 30 31 32 33 34 35 36 37 38 39 40 41 42 43 44 45
##       1001  1  1  1  1  1  1  1  1  1  1  1  1  1  1  1  1  1  1  1  1  1
##       1002  1  1  1  1  1  1  1  1  1  1  1  1  1  1  1  1  1  1  1  1  1
##       1003  1  1  1  1  1  1  1  1  1  1  1  1  1  1  1  1  1  1  1  1  1
##       1004  1  1  1  1  1  1  1  1  1  1  1  1  1  1  1  1  1  1  1  1  1
##       1005  1  1  1  1  1  1  1  1  1  1  1  1  1  1  1  1  1  1  1  1  1
##       1006  1  1  1  1  1  1  1  1  1  1  1  1  1  1  1  1  1  1  1  1  1
##       1008  1  1  1  1  1  1  1  1  1  1  1  1  1  1  1  1  1  1  1  1  1
##       1009  1  1  1  1  1  1  0  0  0  0  0  0  0  0  0  0  0  0  0  0  0
##       1010  1  1  1  1  1  1  1  1  1  1  1  1  1  1  1  1  1  1  1  1  1
##       1011  1  1  1  1  1  1  1  1  1  1  1  1  1  1  1  1  1  1  1  1  1
##       1012  1  1  1  1  1  1  1  1  1  1  1  1  1  1  1  1  1  1  1  1  1
##       1017  1  1  1  1  1  1  1  1  1  1  1  1  1  1  1  1  1  1  1  1  1
##       1019  1  1  1  1  1  1  1  1  1  1  1  1  1  1  1  1  1  1  1  1  1
##       1021  1  1  1  1  1  1  1  1  1  1  1  1  1  1  1  1  1  1  1  1  1
##       1022  1  1  1  1  1  1  1  1  1  1  1  1  1  1  1  1  1  1  1  1  1
##       1023  1  1  1  1  1  1  1  1  1  1  1  1  1  1  1  1  1  1  0  0  0
##       1024  1  1  1  1  1  1  1  1  1  1  1  1  1  1  1  1  1  1  1  1  1
##       1026  1  1  1  1  1  1  1  1  1  1  1  1  1  1  1  1  1  1  1  1  1
##       1028  1  1  1  1  1  1  1  1  1  1  1  1  1  1  1  1  1  1  1  1  1
##       1029  1  1  1  1  1  1  1  1  1  1  1  1  1  1  1  1  1  1  1  1  1
##       1031  1  1  1  1  1  1  1  1  1  1  1  1  1  1  1  1  1  1  1  1  1
##       1033  1  1  1  1  1  1  1  1  1  1  1  1  1  1  1  1  1  1  1  1  1
##       1036  1  1  1  1  1  1  1  1  1  1  1  1  1  1  1  1  1  1  1  1  1
##       1039  1  1  1  1  1  1  1  1  1  1  1  1  1  1  1  1  1  1  1  1  1
##       1040  1  1  1  1  1  1  1  1  1  1  1  1  1  1  1  1  1  1  1  1  1
##       1043  1  1  1  1  1  1  1  1  1  1  1  1  1  1  1  1  1  1  1  1  1
##       1044  0  0  0  0  0  0  0  0  0  0  0  0  0  0  0  0  0  0  0  0  0
##       1045  1  1  1  1  1  1  1  1  1  1  1  1  1  1  1  1  1  1  1  1  1
##       1046  1  1  1  0  0  0  0  0  0  0  0  0  0  0  0  0  0  0  0  0  0
##       1047  1  1  1  1  1  1  1  1  1  1  1  1  1  1  1  1  1  1  1  1  1
##       1048  1  1  1  1  1  1  1  1  1  1  1  1  1  1  1  1  1  1  1  1  1
##       1049  1  1  1  1  1  1  1  1  1  1  1  1  1  1  1  1  1  1  1  1  1
##       1050  1  1  1  1  1  1  1  1  1  1  1  1  1  1  1  1  1  1  1  1  1
##       1051  1  1  1  1  1  1  1  1  1  1  1  1  1  1  1  1  1  1  1  1  1
```

© 2019 Turner NA et al. *JAMA Network Open*

```
##       1052  1  1  1  1  1  1  1  1  1  1  1  1  1  1  1  1  1  1  1  1  1
##       1054  0  0  0  0  0  0  1  1  1  1  1  1  1  1  1  1  1  1  1  1  1
##       1055  0  0  0  0  0  0  0  0  0  0  0  0  1  1  1  1  1  1  1  1  1
##       1056  0  0  0  0  0  0  0  0  0  0  0  0  1  1  1  1  1  1  1  1  1
##       1057  1  1  1  1  1  1  1  1  1  1  1  1  1  1  1  1  1  1  1  1  1
##       1058  0  0  0  0  0  0  0  0  0  0  0  0  1  1  1  1  1  1  1  1  1
##       1060  1  1  1  1  1  1  1  1  1  1  1  1  1  1  1  1  1  1  1  1  1
##       1062  1  1  1  1  1  1  1  1  1  1  1  1  1  1  1  1  1  1  1  1  1
##       1063  1  1  1  1  1  1  1  1  1  1  1  1  1  1  1  1  1  1  1  1  1
##           date3
## HospitalID 46 47 48 49 50 51 52 53 54 55 56 57 58 59 60
##       1001  1  1  1  1  1  1  1  1  1  1  1  1  1  1  1
##       1002  1  1  1  1  1  1  1  1  1  1  1  1  1  1  1
##       1003  1  1  1  1  1  1  1  1  1  1  1  1  1  1  1
##       1004  1  1  1  1  1  1  1  1  1  1  1  1  1  1  1
##       1005  1  1  1  1  1  1  1  1  1  1  1  1  1  1  1
##       1006  1  1  1  1  1  1  1  1  1  1  1  1  1  1  1
##       1008  1  1  1  1  1  1  1  1  1  1  1  1  1  1  1
##       1009  0  0  0  0  0  0  0  0  0  0  0  0  0  0  0
##       1010  1  1  1  1  1  1  1  1  1  1  1  1  1  1  1
##       1011  1  1  1  1  1  1  1  1  1  1  1  1  1  1  1
##       1012  1  1  1  1  1  1  1  1  1  1  1  1  1  1  1
##       1017  1  1  1  1  1  1  1  1  1  1  1  1  1  1  1
##       1019  1  1  1  1  1  1  1  1  1  1  1  1  1  1  1
##       1021  1  1  1  1  1  1  1  1  1  1  1  1  1  1  1
##       1022  1  1  1  1  1  1  1  1  1  1  1  1  1  1  1
##       1023  0  0  0  0  0  0  0  0  0  0  0  0  0  0  0
##       1024  1  1  1  1  1  1  1  1  1  1  1  1  1  1  1
##       1026  1  1  1  1  1  1  1  1  1  1  1  1  1  1  1
##       1028  1  1  1  1  1  1  1  1  1  1  1  1  1  1  1
##       1029  1  1  1  1  1  1  1  1  1  1  1  1  1  1  1
##       1031  1  1  1  1  1  1  1  1  1  1  1  1  1  1  1
##       1033  1  1  1  1  1  1  1  1  1  1  1  1  1  1  1
##       1036  1  1  1  1  1  1  1  1  1  1  1  1  1  1  1
##       1039  1  1  1  1  1  1  1  1  1  1  1  1  1  1  1
##       1040  1  1  1  1  1  1  1  1  1  1  1  1  1  1  1
##       1043  1  1  1  1  1  1  1  1  1  1  1  1  1  1  1
##       1044  0  0  0  0  0  0  0  0  0  0  0  0  0  0  0
##       1045  1  1  1  1  1  1  1  1  1  1  1  1  1  1  1
##       1046  0  0  0  0  0  0  0  0  0  0  0  0  0  0  0
##       1047  1  1  1  1  1  1  1  1  1  1  1  1  1  1  1
##       1048  1  1  1  1  1  1  1  1  1  1  1  1  1  1  1
##       1049  1  1  1  1  1  1  1  1  1  0  0  0  0  0  0
##       1050  1  1  1  1  1  1  1  1  1  1  1  1  1  1  1
##       1051  1  1  1  1  1  1  1  1  1  1  1  1  1  1  1
##       1052  1  1  1  1  1  1  1  1  1  1  1  1  1  1  1
##       1054  1  1  1  1  1  1  1  1  1  1  1  1  1  1  1
##       1055  1  1  1  1  1  1  1  1  1  1  1  1  1  1  1
##       1056  1  1  1  1  1  1  1  1  1  1  0  0  0  0  0
##       1057  1  1  1  1  1  1  1  1  1  1  1  1  1  1  1
##       1058  1  1  1  1  1  1  1  1  1  1  1  1  1  1  1
##       1060  1  1  1  1  1  1  1  1  1  1  1  1  1  1  1
##       1062  1  1  1  1  1  1  1  1  1  1  1  1  1  1  1
##       1063  1  1  1  1  1  1  1  1  1  1  1  1  1  1  1
```

```
print(xyplot(HCFA_CDI_cases ~ date3 | HospitalID, hcfa_cdi_final, aspect = "xy",
             layout = c(5,5), type = c("g", "p", "r"),
             index.cond = function(x,y) coef(lm(y ~ x))[1],
             xlab = "Month",
             ylab = "HCFA cases"))
```

© 2019 Turner NA et al. *JAMA Network Open*

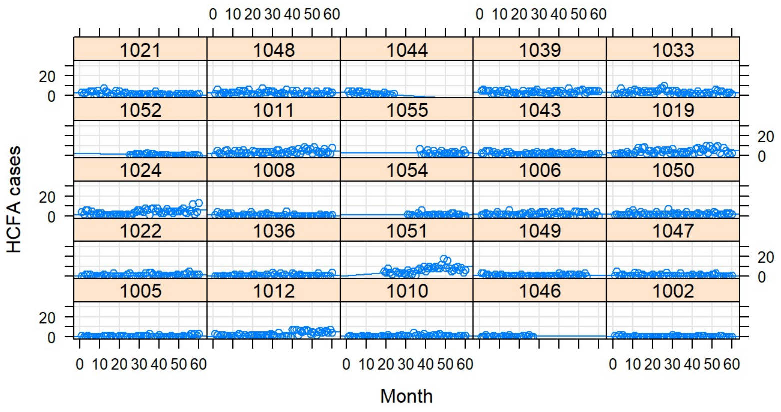


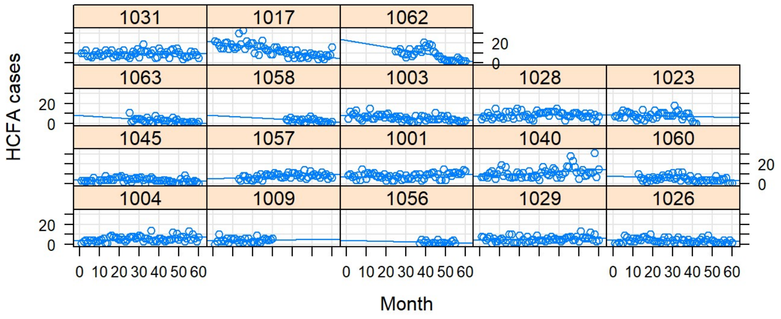


```
## Ib: Examine Data Distributions
library(car)
```

```
## Loading required package: carData
```

```
##
## Attaching package: 'car'
```

```
## The following object is masked from 'package:dplyr':
##
##     recode
```

```
## The following object is masked from 'package:purrr':
##
##     some
```

```
library(MASS)
```

```
##
## Attaching package: 'MASS'
```

© 2019 Turner NA et al. *JAMA Network Open*

```
## The following object is masked from 'package:dplyr':
##
##     select
```

```
# CA CDI
qqp(ca_cdi_final$CA_CDI_cases, "norm")  # Clearly does not fit normal distribution
```

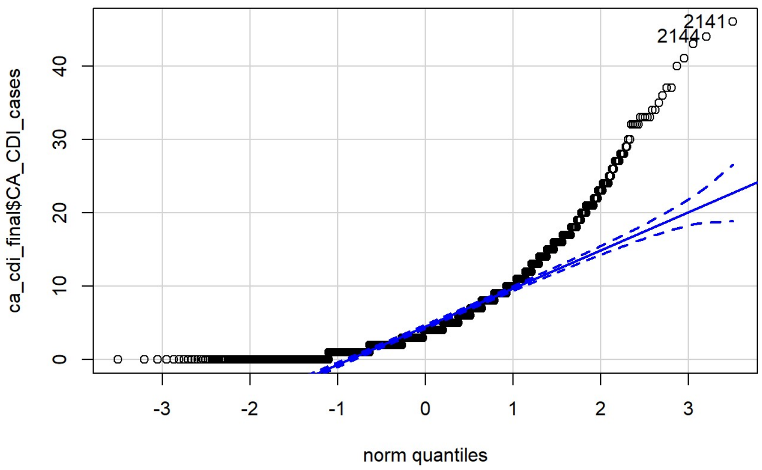


```
## [1] 2141 2144
```

```
poisson <- fitdistr(ca_cdi_final$CA_CDI_cases, "Poisson")  # Lambda estimate: 5.495
poisson
```

```
##     lambda
##   5.5175080
##  (0.0500909)
```

```
qqp(ca_cdi_final$CA_CDI_cases, "pois", poisson$estimate, lambda=5.518)  # Does not fit Poisson
```

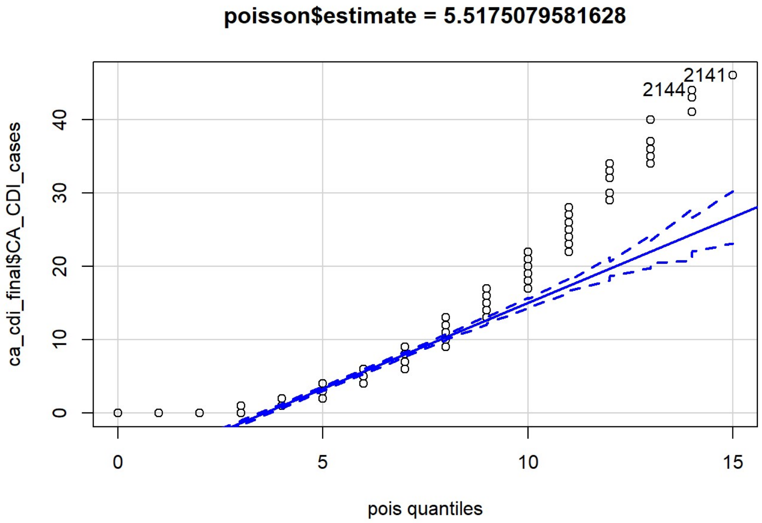


```
nbinom <- fitdistr(ca_cdi_final$CA_CDI_cases, "Negative Binomial")
qqp(ca_cdi_final$CA_CDI_cases, "nbinom", size=nbinom$estimate[[1]], mu=nbinom$estimate[[2]])  # Fits negative binomial rathe
r well
```

© 2019 Turner NA et al. *JAMA Network Open*

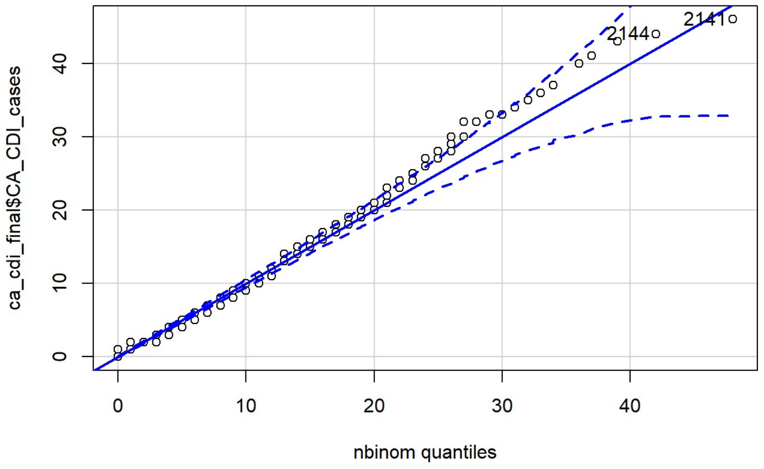


```
## [1] 2141 2144
```

```
# HCFA CDI
qqp(hcfa_cdi_final$HCFA_CDI_cases, "norm")  # Does not fit again
```

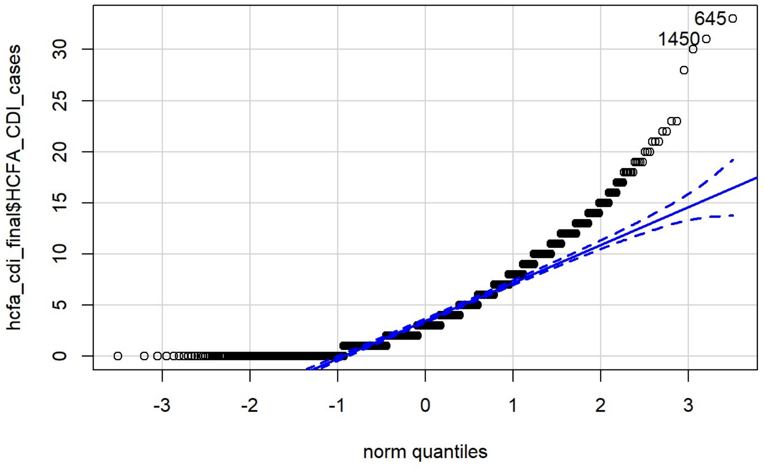


```
## [1]  645 1450
```

```
poisson <- fitdistr(hcfa_cdi_final$HCFA_CDI_cases, "Poisson")  # Lambda estimate: 4.021
poisson
```

```
##      lambda
##   4.03001364
##  (0.04280955)
```

```
qqp(hcfa_cdi_final$HCFA_CDI_cases, "pois", poisson$estimate, lambda=4.030)  # Does not fit again
```

© 2019 Turner NA et al. *JAMA Network Open*

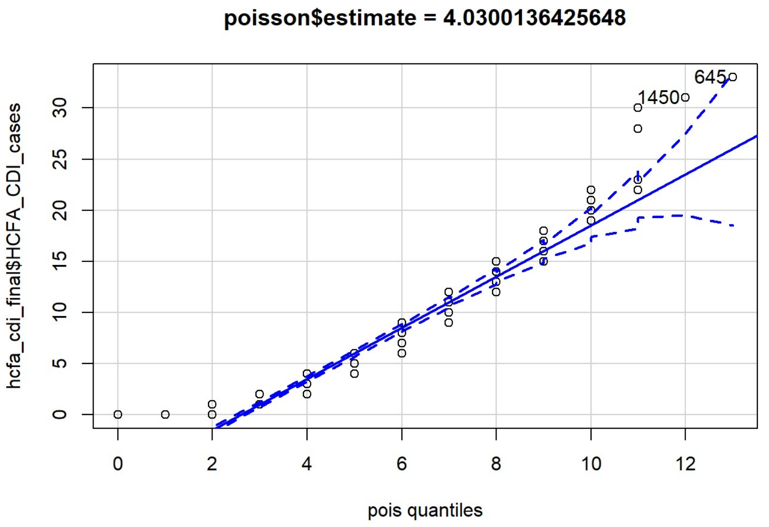


```
nbinom <- fitdistr(hcfa_cdi_final$HCFA_CDI_cases, "Negative Binomial")
```

```
## Warning in densfun(x, parm[1], parm[2], ...): NaNs produced

## Warning in densfun(x, parm[1], parm[2], ...): NaNs produced
```

```
qqp(hcfa_cdi_final$HCFA_CDI_cases, "nbinom", size=nbinom$estimate[[1]], mu=nbinom$estimate[[2]])  # Fits negative binomial w
ell
```

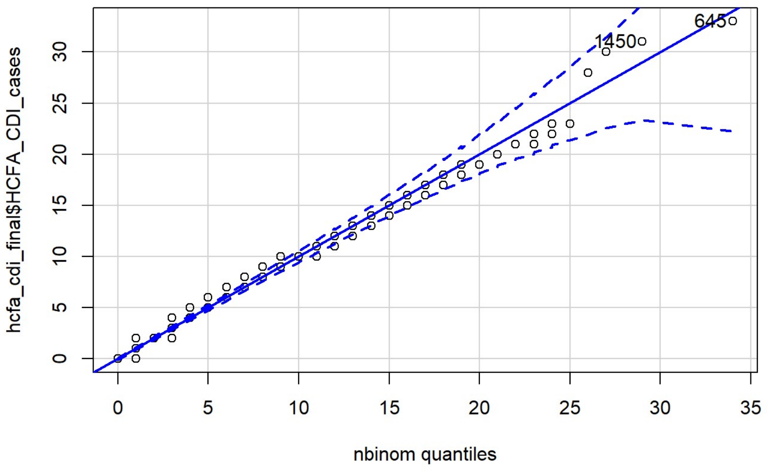


```
## [1]  645 1450
```

```
## Ic: Examine for zero inflation:
hist(ca_cdi_final$CA_CDI_cases, breaks=50)
```

© 2019 Turner NA et al. *JAMA Network Open*

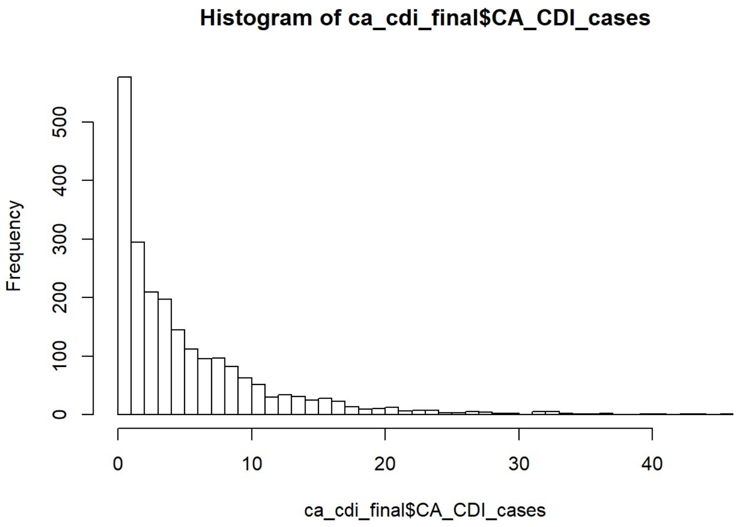


```
hist(hcfa_cdi_final$HCFA_CDI_cases, breaks=50)
```

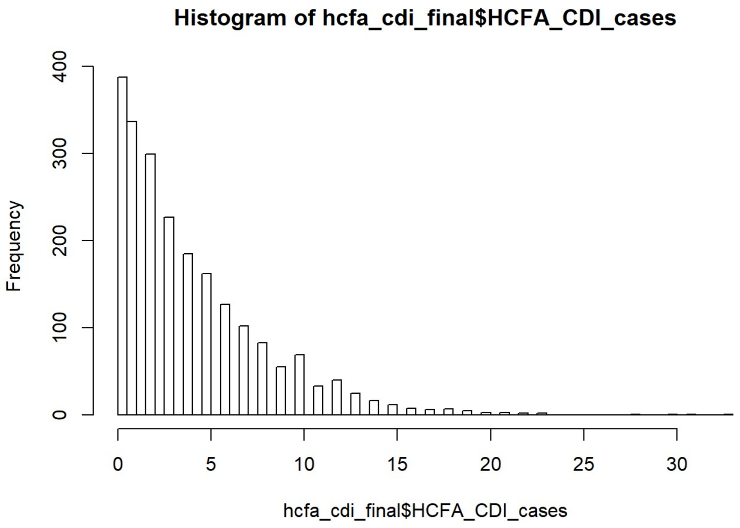


```
### Phase II: Mixed Effects Modeling ###

# Load glmmTMB for modeling:
library(glmmTMB)
```

```
## Registered S3 methods overwritten by 'lme4':
##   method                          from
##   cooks.distance.influence.merMod car
##   influence.merMod                car
##   dfbeta.influence.merMod         car
##   dfbetas.influence.merMod        car
```

© 2019 Turner NA et al. *JAMA Network Open*

```r
library(ggplot2)
theme_set(theme_bw()+
            theme(panel.spacing=grid::unit(0,"lines")))

# Given distributions above, we make the following modeling selections:
# There is evidence of zero inflation on histograms, though turned out non-significant on model testing
# Negative binomial better accounts for the overdispersion
# Random effects: hospitalID and time incuded

# Final model specifications:

## CA CDI:

# CA Model 6: Negative Binomial 2 with correlated random intercept/random slope (date3|HospitalID), no zero inflation compon
ent
ca_model6 <- glmmTMB(CA_CDI_cases~date3+Urban+Academic+Molecular+offset(log(CDiffTotalAdmissions))+(date3|HospitalID), data=
ca_cdi_final, ziformula=~0, family=nbinom2)
summary(ca_model6)
```

```
##  Family: nbinom2  ( log )
## Formula:
## CA_CDI_cases ~ date3 + Urban + Academic + Molecular + offset(log(CDiffTotalAdmissions)) +
##     (date3 | HospitalID)
## Data: ca_cdi_final
##
##      AIC      BIC   logLik deviance df.resid
##   9730.3   9781.5  -4856.1   9712.3     2190
##
## Random effects:
##
## Conditional model:
##  Groups     Name        Variance  Std.Dev. Corr
##  HospitalID (Intercept) 0.4485785 0.66976
##             date3       0.0002207 0.01486  -0.69
## Number of obs: 2199, groups:  HospitalID, 43
##
## Overdispersion parameter for nbinom2 family (): 15.4
##
## Conditional model:
##                Estimate Std. Error z value Pr(>|z|)
## (Intercept)   -5.8066902  0.1765971  -32.88   <2e-16 ***
## date3          0.0037558  0.0025645    1.46    0.143
## Urban         -0.1480356  0.1655180   -0.89    0.371
## AcademicMinor -0.0008344  0.1544558   -0.01    0.996
## Molecular      0.6517422  0.0762464    8.55   <2e-16 ***
## ---
## Signif. codes:  0 '***' 0.001 '**' 0.01 '*' 0.05 '.' 0.1 ' ' 1
```

```r
exp(confint(ca_model6))
```

```
##                                  2.5 %       97.5 %     Estimate
## cond.(Intercept)          2.127486e-03 4.251148e-03 3.007367e-03
## cond.date3                9.987303e-01 1.008821e+00 1.003763e+00
## cond.Urban                6.234760e-01 1.192884e+00 8.624004e-01
## cond.AcademicMinor        7.381840e-01 1.352417e+00 9.991660e-01
## cond.Molecular            1.652521e+00 2.228174e+00 1.918881e+00
## cond.Std.Dev.(Intercept)  1.662355e+00 2.417224e+00 1.953768e+00
## cond.Std.Dev.date3        1.011186e+00 1.020039e+00 1.014967e+00
## cond.Cor.date3.(Intercept) 4.359885e-01 6.692931e-01 4.996227e-01
## sigma                     1.857939e+05 2.948207e+08 4.789576e+06
```

```r
# Check residuals: All reasonably well behaved
plot(fitted(ca_model6,type="response"), residuals(ca_model6))
```

© 2019 Turner NA et al. *JAMA Network Open*

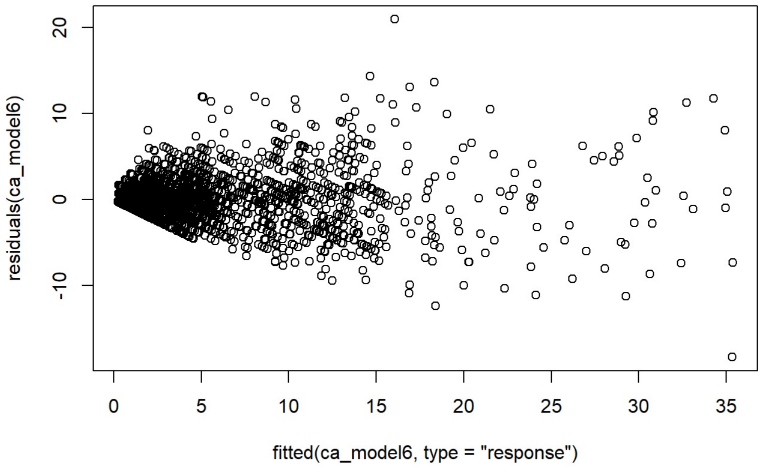


```
# Comparing actual/predicted:
ca_cdi_final$predicted <- predict(ca_model6, ca_cdi_final)
ca_cdi_final$predicted_rate <- exp(ca_cdi_final$predicted)/ca_cdi_final$CDiffTotalAdmissions*1000

actual_v_fitted_ca <- ggplot(ca_cdi_final, aes(x=ca_cdi_final$incidence_a, y=ca_cdi_final$predicted_rate))+
  geom_point()+
  geom_abline(intercept=0, slope=1, color="red")+
  ggtitle("Actual vs Fitted Incidence Rates for CA-CDI")+xlab("Actual")+ ylab("Fitted")
actual_v_fitted_ca
```

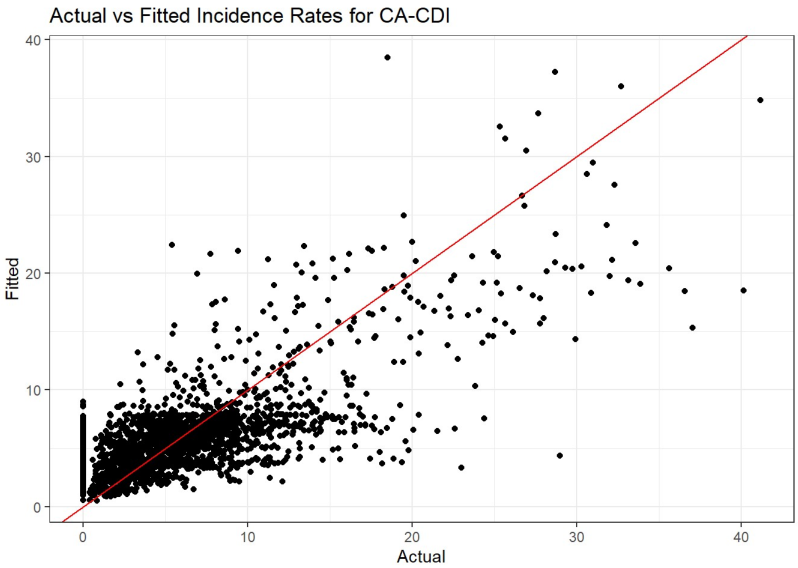


```
# Predictions: typical urban community hospital
mean(ca_cdi_final$CDiffTotalAdmissions) # 940.8
```

```
## [1] 940.8445
```

```
range(ca_cdi_final$date3) # 1:60
```

```
## [1]  1 60
```

```
# Load empty dataset (including only date indicators, mean total admissions, modal hospital attributes)
ndata <- read_csv("P:/DICON_OUTREACH/Research/Nick Turner ARLG Research/DICON CDI Basic Epi/ndata.csv")
```

© 2019 Turner NA et al. *JAMA Network Open*

```
## Parsed with column specification:
## cols(
##   date3 = col_double(),
##   Urban = col_double(),
##   Academic = col_character(),
##   Molecular = col_double(),
##   CDiffTotalAdmissions = col_double(),
##   HospitalID = col_character()
## )
```

```
ndata$CDiffTotalAdmissions <- 941

# Organizing a fitted response:
pred_ca <- cbind(ndata, "model"=predict(ca_model6, newdata=ndata, type="link", se.fit=TRUE, allow.new.levels=TRUE))
pred_ca$predicted_rate <- exp(pred_ca$model.fit)/941*1000
pred_ca$ll <- exp((pred_ca$model.fit)-1.96*(pred_ca$model.se.fit))/941*1000
pred_ca$ul <- exp((pred_ca$model.fit)+1.96*(pred_ca$model.se.fit))/941*1000
pred_ca$Molecular <- as.factor(pred_ca$Molecular)

# Plotting CA predicted (stratified by test type):
ca_pred_plot <- ggplot(pred_ca, aes(x=date3, y=predicted_rate, group=Molecular, color=Molecular))+
  geom_line(size=2)+
  geom_ribbon(aes(ymin=ll, ymax=ul, group=Molecular, fill=Molecular), alpha=0.15)+
  scale_x_continuous(name="Year", breaks=c(1, 13, 25, 37, 49), labels=c("2013", "2014", "2015", "2016", "2017"))+
  ylab("Modeled CA-CDI Rate (per 1,000 admissions)")+
  labs(NULL)+
  ylim(0,30)
ca_pred_plot
```

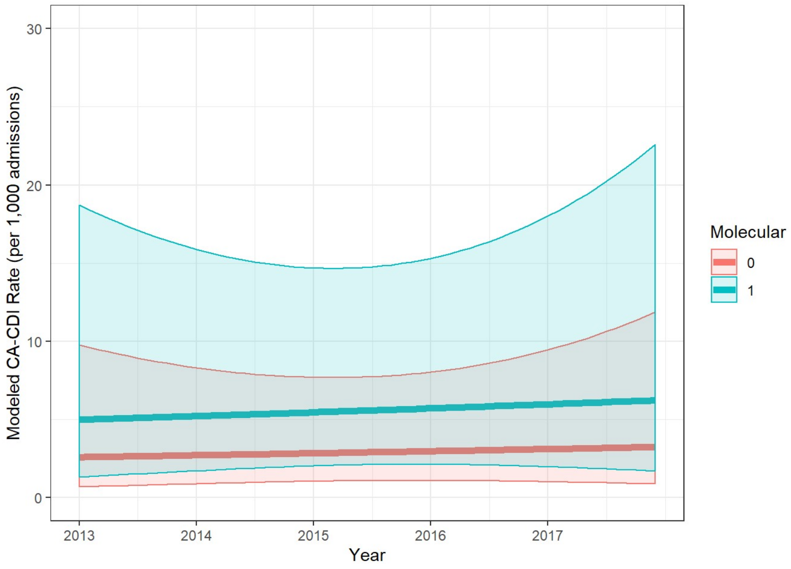


```
# Plotting CA predicted (presuming molecular testing):
pred_ca_molec <- subset(pred_ca, pred_ca$Molecular==1)

ca_pred_plot2 <- ggplot(pred_ca_molec, aes(x=date3, y=predicted_rate))+
  geom_line()+
  geom_ribbon(aes(ymin=ll, ymax=ul), alpha=0.25)+
  scale_x_continuous(name="Year", breaks=c(1, 13, 25, 37, 49), labels=c("2013", "2014", "2015", "2016", "2017"))+
  ylab("Modeled CA-CDI Rate (per 1,000 admissions)")+
  labs(NULL)+
  ylim(0,30)
ca_pred_plot2
```

© 2019 Turner NA et al. *JAMA Network Open*

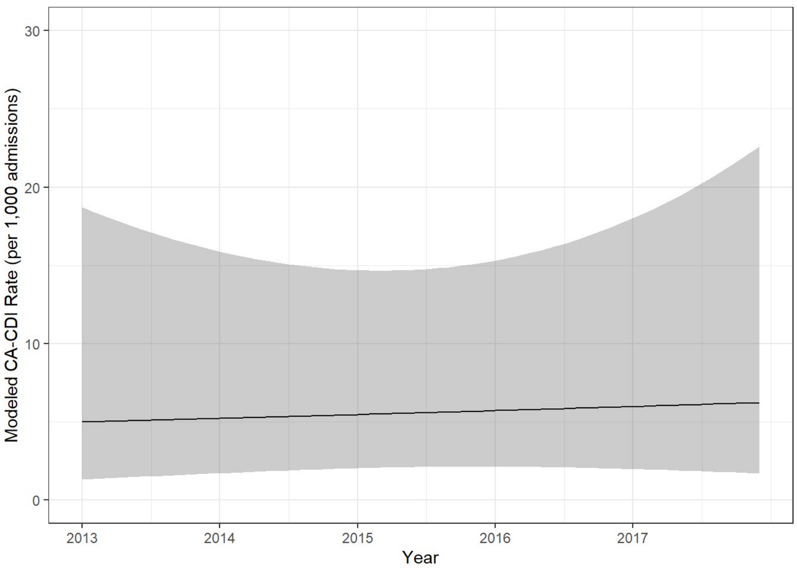


```
# CA Model 6: Negative Binomial 2 with correlated random intercept/random slope (date3|HospitalID), no zero inflation compon
ent
# Repeating but unadjusted:
ca_model6_un <- glmmTMB(CA_CDI_cases~date3+offset(log(CDiffTotalAdmissions))+(date3|HospitalID), data=ca_cdi_final, ziformul
a=~0, family=nbinom2)
summary(ca_model6_un)
```

```
##  Family: nbinom2  ( log )
## Formula:
## CA_CDI_cases ~ date3 + offset(log(CDiffTotalAdmissions)) + (date3 |
##     HospitalID)
## Data: ca_cdi_final
##
##      AIC      BIC   logLik deviance df.resid
##   9797.6   9831.7  -4892.8   9785.6     2193
##
## Random effects:
##
## Conditional model:
##  Groups     Name        Variance  Std.Dev. Corr
##  HospitalID (Intercept) 0.6533024 0.80827
##             date3       0.0003242 0.01801  -0.77
## Number of obs: 2199, groups:  HospitalID, 43
##
## Overdispersion parameter for nbinom2 family ():   14
##
## Conditional model:
##              Estimate Std. Error z value Pr(>|z|)
## (Intercept) -5.519898   0.131218  -42.07   <2e-16 ***
## date3        0.007356   0.002990    2.46   0.0139 *
## ---
## Signif. codes:  0 '***' 0.001 '**' 0.01 '*' 0.05 '.' 0.1 ' ' 1
```

```
exp(confint(ca_model6_un))
```

```
##                                   2.5 %       97.5 %     Estimate
## cond.(Intercept)           3.097744e-03 5.181218e-03 4.006256e-03
## cond.date3                 1.001496e+00 1.013305e+00 1.007383e+00
## cond.Std.Dev.(Intercept)   1.863617e+00 2.856079e+00 2.244025e+00
## cond.Std.Dev.date3         1.013831e+00 1.023886e+00 1.018170e+00
## cond.Cor.date3.(Intercept) 4.200169e-01 5.833966e-01 4.648585e-01
## sigma                      6.950474e+04 3.990428e+07 1.165870e+06
```

```
## Repeat modeling for HCFA:

# HCFA Model 6: Negative Binomial 2 with correlated random intercept/random slope (date3|HospitalID), no zero inflation comp
onent
hcfa_model6 <- glmmTMB(HCFA_CDI_cases~date3+Urban+Academic+Molecular+offset(log(CDiffPatientDays))+(date3|HospitalID), data=
hcfa_cdi_final, ziformula=~0, family=nbinom2)
summary(hcfa_model6)
```

© 2019 Turner NA et al. *JAMA Network Open*

```
##  Family: nbinom2  ( log )
## Formula:
## HCFA_CDI_cases ~ date3 + Urban + Academic + Molecular + offset(log(CDiffPatientDays)) +
##     (date3 | HospitalID)
## Data: hcfa_cdi_final
##
##      AIC      BIC   logLik deviance df.resid
##   8663.9   8715.2  -4323.0   8645.9     2190
##
## Random effects:
##
## Conditional model:
##  Groups     Name        Variance  Std.Dev. Corr
##  HospitalID (Intercept) 0.2170958 0.4659
##             date3       0.0001988 0.0141   -0.69
## Number of obs: 2199, groups:  HospitalID, 43
##
## Overdispersion parameter for nbinom2 family (): 23.4
##
## Conditional model:
##                Estimate Std. Error z value Pr(>|z|)
## (Intercept)   -7.157157   0.132381  -54.06   <2e-16 ***
## date3         -0.005401   0.002475   -2.18   0.0291 *
## Urban         -0.220142   0.122363   -1.80   0.0720 .
## AcademicMinor  0.025425   0.112285    0.23   0.8209
## Molecular      0.604925   0.071771    8.43   <2e-16 ***
## ---
## Signif. codes:  0 '***' 0.001 '**' 0.01 '*' 0.05 '.' 0.1 ' ' 1
```

```
exp(confint(hcfa_model6))
```

```
##                                  2.5 %       97.5 %     Estimate
## cond.(Intercept)           6.011774e-04 1.010111e-03 7.792665e-04
## cond.date3                 9.898010e-01 9.994501e-01 9.946139e-01
## cond.Urban                 6.313030e-01 1.019881e+00 8.024052e-01
## cond.AcademicMinor         8.231234e-01 1.278260e+00 1.025751e+00
## cond.Molecular             1.590831e+00 2.107693e+00 1.831115e+00
## cond.Std.Dev.(Intercept)   1.418426e+00 1.860931e+00 1.593504e+00
## cond.Std.Dev.date3         1.010747e+00 1.018775e+00 1.014201e+00
## cond.Cor.date3.(Intercept) 4.357118e-01 6.690760e-01 4.992416e-01
## sigma                      1.139889e+07 4.497807e+14 1.474980e+10
```

```
# Checking residuals: All reasonably well behaved
plot(fitted(hcfa_model6,type="response"), residuals(hcfa_model6))
```

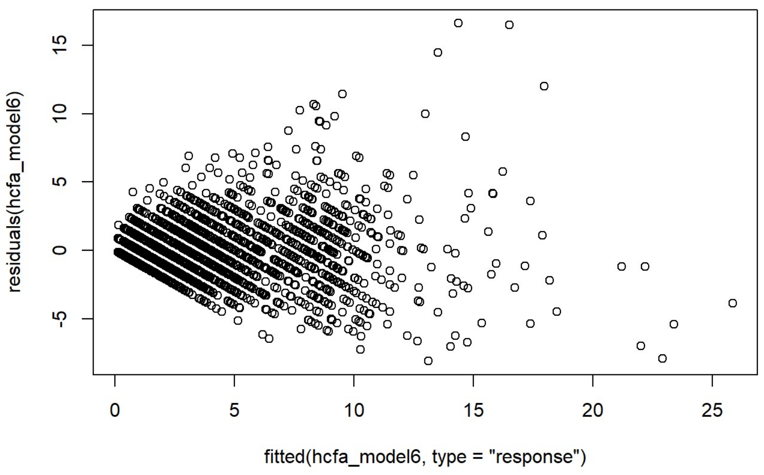


© 2019 Turner NA et al. *JAMA Network Open*

```
# Comparing actual/predicted:
hcfa_cdi_final$predicted <- predict(hcfa_model6, hcfa_cdi_final)
hcfa_cdi_final$predicted_rate <- exp(hcfa_cdi_final$predicted)/hcfa_cdi_final$CDiffPatientDays*10000

actual_v_fitted_hcfa <- ggplot(hcfa_cdi_final, aes(x=hcfa_cdi_final$incidence, y=hcfa_cdi_final$predicted_rate))+
  geom_point()+
  geom_abline(intercept=0, slope=1, color="red")+
  ggtitle("Actual vs Fitted Incidence Rates for HCFA-CDI")+xlab("Actual")+ ylab("Fitted")+
  xlim(0,40)+
  ylim(0,40)
actual_v_fitted_hcfa
```

```
## Warning: Removed 17 rows containing missing values (geom_point).
```

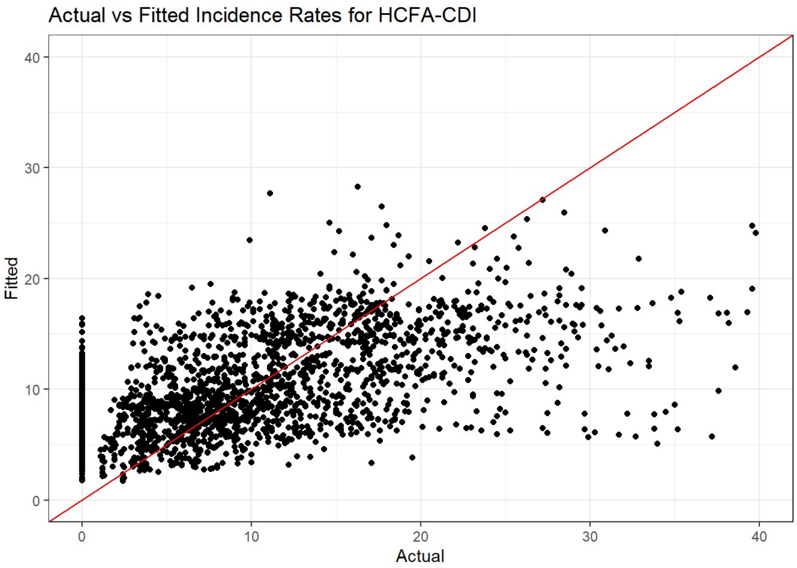


```
# Predictions: typical urban community hospital
mean(hcfa_cdi_final$CDiffTotalAdmissions) # 940.8
```

```
## [1] 940.8445
```

```
median(hcfa_cdi_final$CDiffTotalAdmissions) # median 775
```

```
## [1] 775
```

```
range(hcfa_cdi_final$date3) # 1:60
```

```
## [1]  1 60
```

```
mean(hcfa_cdi_final$CDiffPatientDays) # 4078
```

```
## [1] 4077.618
```

© 2019 Turner NA et al. *JAMA Network Open*

```
# Add patient days to ndata:
ndata$CDiffPatientDays <- 4078

# Organizing a fitted response:
pred_hcfa <- cbind(ndata, "model"=predict(hcfa_model6, newdata=ndata, type="link", se.fit=TRUE, allow.new.levels=TRUE))
pred_hcfa$predicted_rate <- exp(pred_hcfa$model.fit)/4078*10000
pred_hcfa$ll <- exp((pred_hcfa$model.fit)-1.96*(pred_hcfa$model.se.fit))/4078*10000
pred_hcfa$ul <- exp((pred_hcfa$model.fit)+1.96*(pred_hcfa$model.se.fit))/4078*10000
pred_hcfa$Molecular <- as.factor(pred_hcfa$Molecular)

# Ribbon plot:
hcfa_pred_plot <- ggplot(pred_hcfa, aes(x=date3, y=predicted_rate, group=Molecular, color=Molecular))+
  geom_line(size=2)+
  geom_ribbon(aes(ymin=ll, ymax=ul, group=Molecular, fill=Molecular), alpha=0.15)+
  scale_x_continuous(name="Year", breaks=c(1, 13, 25, 37, 49), labels=c("2013", "2014", "2015", "2016", "2017"))+
  ylab("Modeled HCFA-CDI Rate (per 10,000 patient-days)")+
  labs(NULL)+
  ylim(0,30)
hcfa_pred_plot
```

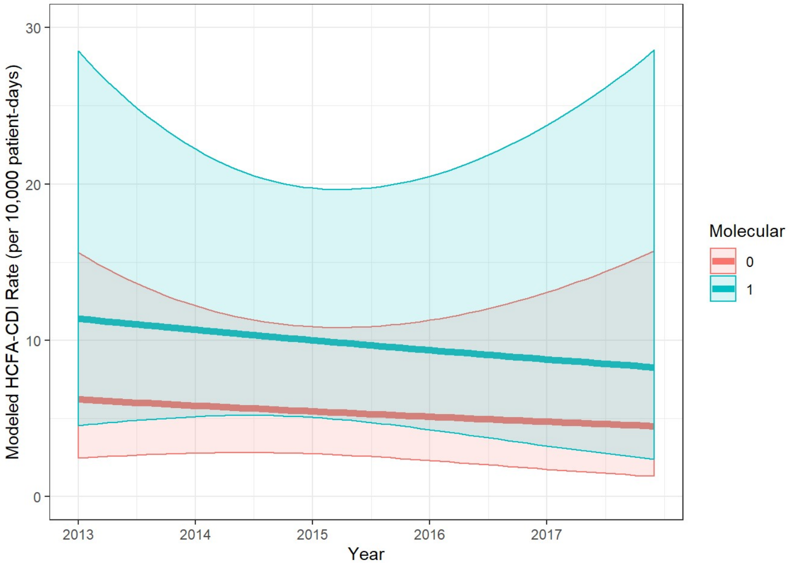


```
# Plotting HCFA predicted (presuming molecular testing):
pred_hcfa_molec <- subset(pred_hcfa, pred_hcfa$Molecular==1)

hcfa_pred_plot2 <- ggplot(pred_hcfa_molec, aes(x=date3, y=predicted_rate))+
  geom_line()+
  geom_ribbon(aes(ymin=ll, ymax=ul), alpha=0.25)+
  scale_x_continuous(name="Year", breaks=c(1, 13, 25, 37, 49), labels=c("2013", "2014", "2015", "2016", "2017"))+
  ylab("Modeled HCFA-CDI Rate (per 10,000 patient-days)")+
  labs(NULL)+
  ylim(0,30)
hcfa_pred_plot2
```

© 2019 Turner NA et al. *JAMA Network Open*

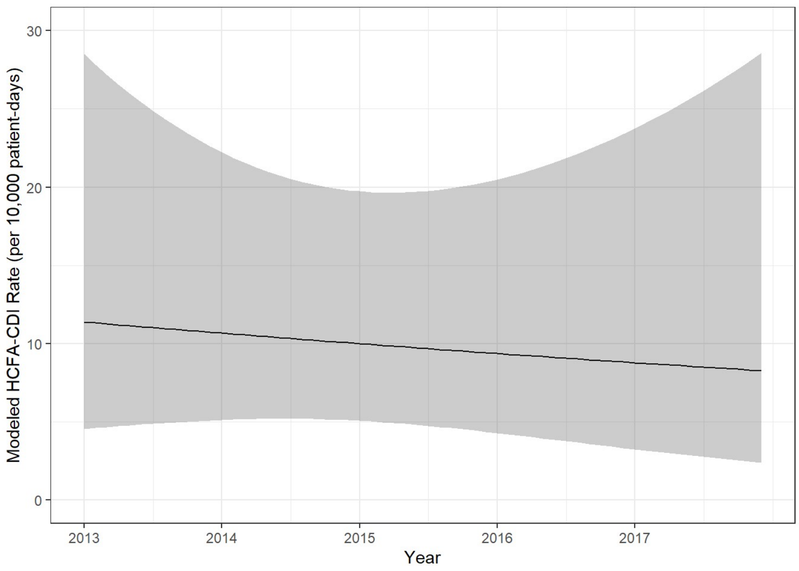


```
# HCFA Model 6: Negative Binomial 2 with correlated random intercept/random slope (date3|HospitalID), no zero inflation comp
onent
# Unadjusted modeling
hcfa_model6_un <- glmmTMB(HCFA_CDI_cases~date3+offset(log(CDiffPatientDays))+(date3|HospitalID), data=hcfa_cdi_final, ziform
ula=~0, family=nbinom2)
summary(hcfa_model6_un)
```

```
##  Family: nbinom2  ( log )
## Formula:
## HCFA_CDI_cases ~ date3 + offset(log(CDiffPatientDays)) + (date3 |
##     HospitalID)
## Data: hcfa_cdi_final
##
##      AIC      BIC   logLik deviance df.resid
##   8729.0   8763.1  -4358.5   8717.0     2193
##
## Random effects:
##
## Conditional model:
##  Groups     Name        Variance  Std.Dev. Corr
##  HospitalID (Intercept) 0.3309040 0.57524
##             date3       0.0002668 0.01633  -0.73
## Number of obs: 2199, groups:  HospitalID, 43
##
## Overdispersion parameter for nbinom2 family (): 21.1
##
## Conditional model:
##              Estimate Std. Error z value Pr(>|z|)
## (Intercept) -6.940396   0.097980  -70.84   <2e-16 ***
## date3       -0.001921   0.002769   -0.69    0.488
## ---
## Signif. codes:  0 '***' 0.001 '**' 0.01 '*' 0.05 '.' 0.1 ' ' 1
```

```
exp(confint(hcfa_model6_un))
```

```
##                                   2.5 %       97.5 %     Estimate
## cond.(Intercept)           7.987737e-04 1.172801e-03 9.678858e-04
## cond.date3                 9.926791e-01 1.003512e+00 9.980808e-01
## cond.Std.Dev.(Intercept)   1.546842e+00 2.135240e+00 1.777562e+00
## cond.Std.Dev.date3         1.012579e+00 1.021570e+00 1.016467e+00
## cond.Cor.date3.(Intercept) 4.292754e-01 6.249827e-01 4.834044e-01
## sigma                      3.218919e+06 8.726960e+12 1.502775e+09
```

© 2019 Turner NA et al. *JAMA Network Open*

```
### Subanalysis by Hospital Testing Strategies

# Assessment of trends over time in hospitals which did not change test type:
# Create exclusion list for hospitals that changed:

# By CA-CDI:
ca_unchanged <- as.data.frame(aggregate(ca_cdi_final$Molecular, list(ca_cdi_final$HospitalID), mean)) %>% filter(x==1|x==0)
ca_changed <- as.data.frame(aggregate(ca_cdi_final$Molecular, list(ca_cdi_final$HospitalID), mean)) %>% filter(x>0 & x<1)

ca_unchanged_list <- ca_unchanged$Group.1
ca_unchanged_cohort <- ca_cdi_final %>% filter(ca_cdi_final$HospitalID %in% ca_unchanged_list)

interaction.plot(x.factor = ca_unchanged_cohort [["date2"]], trace.factor=ca_unchanged_cohort [["HospitalIDLabel"]], respons
e=ca_unchanged_cohort [["incidence_a"]], type="l", legend=FALSE, col=1:60, main="CA-CDI Incidence (per 1,000 admissions)", x
lab="Month", ylab="Incidence per 1,000 admits")
axis(side = 1, at=1:60, labels=FALSE)
```

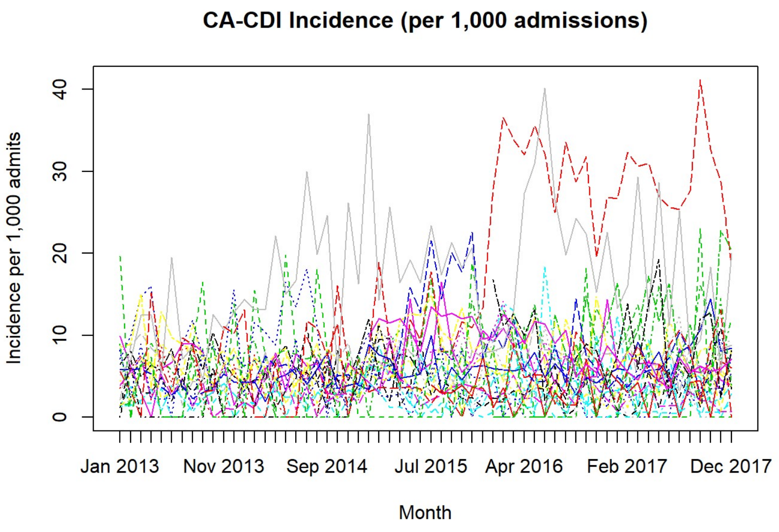


```
ca_model6_unch <- glmmTMB(CA_CDI_cases~date3+offset(log(CDiffTotalAdmissions))+(date3|HospitalID), data=ca_unchanged_cohort,
ziformula=~0, family=nbinom2)
summary(ca_model6_unch)
```

```
##  Family: nbinom2  ( log )
## Formula:
## CA_CDI_cases ~ date3 + offset(log(CDiffTotalAdmissions)) + (date3 |
##     HospitalID)
## Data: ca_unchanged_cohort
##
##      AIC      BIC   logLik deviance df.resid
##   6668.0   6699.6  -3328.0   6656.0     1437
##
## Random effects:
##
## Conditional model:
##  Groups     Name        Variance  Std.Dev. Corr
##  HospitalID (Intercept) 0.3582780 0.59856
##             date3       0.0002394 0.01547  -0.60
## Number of obs: 1443, groups:  HospitalID, 30
##
## Overdispersion parameter for nbinom2 family (): 16.6
##
## Conditional model:
##              Estimate Std. Error z value Pr(>|z|)
## (Intercept) -5.2788548  0.1218911  -43.31   <2e-16 ***
## date3        0.0008693  0.0031856    0.27    0.785
## ---
## Signif. codes:  0 '***' 0.001 '**' 0.01 '*' 0.05 '.' 0.1 ' ' 1
```

```
exp(confint(ca_model6_unch))
```

© 2019 Turner NA et al. *JAMA Network Open*

```
##                                 2.5 %       97.5 %     Estimate
## cond.(Intercept)           4.014843e-03 6.474055e-03 5.098266e-03
## cond.date3                 9.946400e-01 1.007138e+00 1.000870e+00
## cond.Std.Dev.(Intercept)   1.507190e+00 2.394855e+00 1.819503e+00
## cond.Std.Dev.date3         1.010865e+00 1.022402e+00 1.015593e+00
## cond.Cor.date3.(Intercept) 4.472564e-01 8.743094e-01 5.501352e-01
## sigma                      3.158969e+05 2.889670e+09 1.633833e+07
```

```
ca_changed_list <- ca_changed$Group.1
ca_changed_cohort <- ca_cdi_final %>% filter(ca_cdi_final$HospitalID %in% ca_changed_list)

interaction.plot(x.factor = ca_changed_cohort [["date2"]], trace.factor=ca_changed_cohort [["HospitalIDLabel"]], response=ca
_changed_cohort [["incidence_a"]], type="l", legend=FALSE, col=1:60, main="CA-CDI Incidence (per 1,000 admissions)", xlab="M
onth", ylab="Incidence per 1,000 admits")
axis(side = 1, at=1:60, labels=FALSE)
```

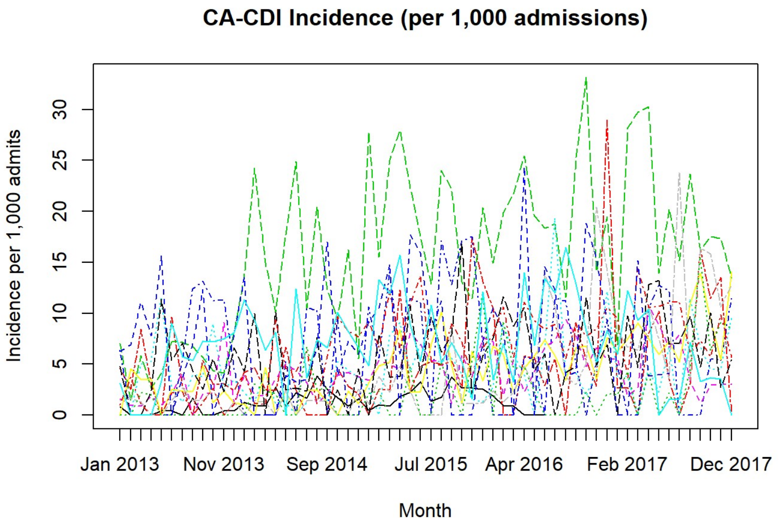


```
ca_model6_cha <- glmmTMB(CA_CDI_cases~date3+offset(log(CDiffTotalAdmissions))+(date3|HospitalID), data=ca_changed_cohort, zi
formula=~0, family=nbinom2)
summary(ca_model6_cha)
```

```
##  Family: nbinom2  ( log )
## Formula:
## CA_CDI_cases ~ date3 + offset(log(CDiffTotalAdmissions)) + (date3 |
##     HospitalID)
## Data: ca_changed_cohort
##
##      AIC      BIC   logLik deviance df.resid
##   3116.4   3144.1  -1552.2   3104.4      750
##
## Random effects:
##
## Conditional model:
##  Groups     Name        Variance  Std.Dev. Corr
##  HospitalID (Intercept) 0.7561711 0.86958
##             date3       0.0001837 0.01355  -0.86
## Number of obs: 756, groups:  HospitalID, 13
##
## Overdispersion parameter for nbinom2 family ():  7.8
##
## Conditional model:
##              Estimate Std. Error z value Pr(>|z|)
## (Intercept) -6.009696   0.249487 -24.088  < 2e-16 ***
## date3        0.020729   0.004144   5.003 5.66e-07 ***
## ---
## Signif. codes:  0 '***' 0.001 '**' 0.01 '*' 0.05 '.' 0.1 ' ' 1
```

```
exp(confint(ca_model6_cha))
```

© 2019 Turner NA et al. *JAMA Network Open*

```
##                                 2.5 %       97.5 %     Estimate
## cond.(Intercept)           1.505422e-03 4.003008e-03 2.454835e-03
## cond.date3                 1.012687e+00 1.029271e+00 1.020945e+00
## cond.Std.Dev.(Intercept)   1.772757e+00 3.746192e+00 2.385911e+00
## cond.Std.Dev.date3         1.008494e+00 1.021959e+00 1.013647e+00
## cond.Cor.date3.(Intercept) 3.882558e-01 6.896584e-01 4.245784e-01
## sigma                      2.272127e+02 7.331578e+04 2.431802e+03
```

```
# By HCFA-CDI:
hcfa_unchanged <- as.data.frame(aggregate(hcfa_cdi_final$Molecular, list(hcfa_cdi_final$HospitalID), mean)) %>% filter(x==1|
x==0)
hcfa_changed <- as.data.frame(aggregate(hcfa_cdi_final$Molecular, list(hcfa_cdi_final$HospitalID), mean)) %>% filter(x>0 & x
<1)

hcfa_unchanged_list <- hcfa_unchanged$Group.1
hcfa_unchanged_cohort <- hcfa_cdi_final %>% filter(hcfa_cdi_final$HospitalID %in% hcfa_unchanged_list)

interaction.plot(x.factor = hcfa_unchanged_cohort [["date2"]], trace.factor=hcfa_unchanged_cohort [["HospitalIDLabel"]], res
ponse=hcfa_unchanged_cohort [["incidence_a"]], type="l", legend=FALSE, col=1:60, main="HCFA-CDI Incidence (per 10,000 patien
t days)", xlab="Month", ylab="Incidence per 10,000 patient days")
axis(side = 1, at=1:60, labels=FALSE)
```

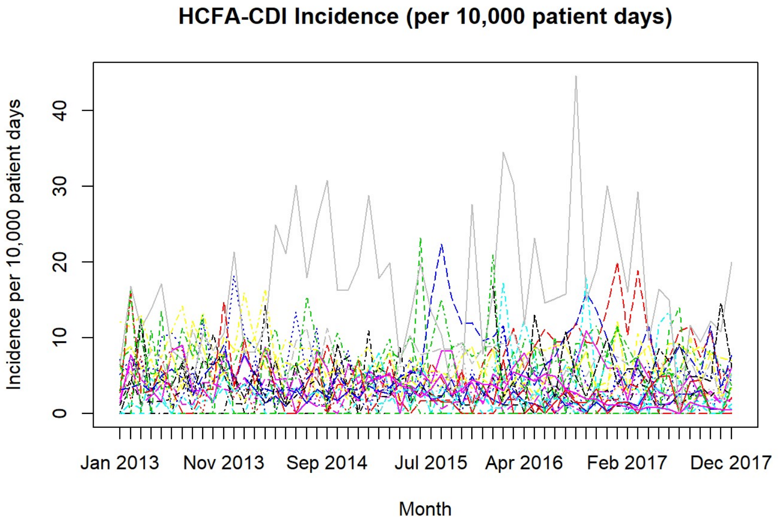


```
hcfa_model6_unch <- glmmTMB(HCFA_CDI_cases~date3+offset(log(CDiffPatientDays))+(date3|HospitalID), data=hcfa_unchanged_cohor
t, ziformula=~0, family=nbinom2)
summary(hcfa_model6_unch)
```

```
##  Family: nbinom2  ( log )
## Formula:
## HCFA_CDI_cases ~ date3 + offset(log(CDiffPatientDays)) + (date3 |
##     HospitalID)
## Data: hcfa_unchanged_cohort
##
##      AIC      BIC   logLik deviance df.resid
##   5895.8   5927.5  -2941.9   5883.8     1437
##
## Random effects:
##
## Conditional model:
##  Groups     Name        Variance  Std.Dev. Corr
##  HospitalID (Intercept) 0.2762661 0.52561
##             date3       0.0002378 0.01542  -0.64
## Number of obs: 1443, groups:  HospitalID, 30
##
## Overdispersion parameter for nbinom2 family (): 24.5
##
## Conditional model:
##              Estimate Std. Error z value Pr(>|z|)
## (Intercept) -6.782474   0.110596  -61.33   <2e-16 ***
## date3       -0.007425   0.003214   -2.31   0.0209 *
## ---
## Signif. codes:  0 '***' 0.001 '**' 0.01 '*' 0.05 '.' 0.1 ' ' 1
```

© 2019 Turner NA et al. *JAMA Network Open*

```
exp(confint(hcfa_model6_unch))
```

```
##                                  2.5 %       97.5 %     Estimate
## cond.(Intercept)           9.125773e-04 1.407822e-03 1.133467e-03
## cond.date3                 9.863697e-01 9.988751e-01 9.926027e-01
## cond.Std.Dev.(Intercept)   1.452082e+00 2.097326e+00 1.691491e+00
## cond.Std.Dev.date3         1.011103e+00 1.021768e+00 1.015540e+00
## cond.Cor.date3.(Intercept) 4.408664e-01 7.860724e-01 5.261545e-01
## sigma                      1.048662e+07 1.531107e+16 4.567638e+10
```

```
hcfa_changed_list <- hcfa_changed$Group.1
hcfa_changed_cohort <- hcfa_cdi_final %>% filter(hcfa_cdi_final$HospitalID %in% hcfa_changed_list)

interaction.plot(x.factor = hcfa_changed_cohort [["date2"]], trace.factor=hcfa_changed_cohort [["HospitalIDLabel"]], respons
e=hcfa_changed_cohort [["incidence_a"]], type="l", legend=FALSE, col=1:60, main="HCFA-CDI Incidence (per 10,000 patient day
s)", xlab="Month", ylab="Incidence per 10,000 patient days")
axis(side = 1, at=1:60, labels=FALSE)
```

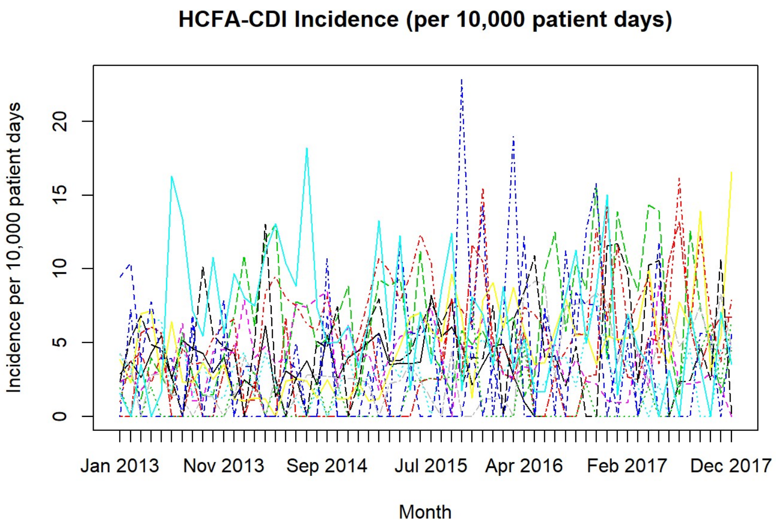


```
hcfa_model6_cha <- glmmTMB(HCFA_CDI_cases~date3+offset(log(CDiffPatientDays))+(date3|HospitalID), data=hcfa_changed_cohort,
ziformula=~0, family=nbinom2)
summary(hcfa_model6_cha)
```

```
##  Family: nbinom2  ( log )
## Formula:
## HCFA_CDI_cases ~ date3 + offset(log(CDiffPatientDays)) + (date3 |
##     HospitalID)
## Data: hcfa_changed_cohort
##
##      AIC      BIC   logLik deviance df.resid
##   2831.5   2859.3  -1409.7   2819.5      750
##
## Random effects:
##
## Conditional model:
##  Groups     Name        Variance  Std.Dev. Corr
##  HospitalID (Intercept) 0.3105530 0.55727
##             date3       0.0001487 0.01219  -0.76
## Number of obs: 756, groups:  HospitalID, 13
##
## Overdispersion parameter for nbinom2 family ():   13
##
## Conditional model:
##              Estimate Std. Error z value Pr(>|z|)
## (Intercept) -7.265563   0.168105  -43.22   <2e-16 ***
## date3        0.009565   0.003828    2.50   0.0125 *
## ---
## Signif. codes:  0 '***' 0.001 '**' 0.01 '*' 0.05 '.' 0.1 ' ' 1
```

```
exp(confint(hcfa_model6_cha))
```

© 2019 Turner NA et al. *JAMA Network Open*

```
##                                   2.5 %       97.5 %     Estimate
## cond.(Intercept)           5.029379e-04 9.720699e-04 6.992072e-04
## cond.date3                 1.002065e+00 1.017214e+00 1.009611e+00
## cond.Std.Dev.(Intercept)   1.408532e+00 2.475882e+00 1.745905e+00
## cond.Std.Dev.date3         1.007599e+00 1.019832e+00 1.012267e+00
## cond.Cor.date3.(Intercept) 4.034518e-01 8.568558e-01 4.689126e-01
## sigma                      1.863112e+03 6.596321e+09 4.641922e+05
```

```
### Logistic regression model of proportion CA-CDI cases over time ###
# Merge data to assess total trends:
# Note: need to exclude sites with 0 cases for a given date as proportion is undefined for these sites:
total_cdi <- left_join(ca_cdi_final, hcfa_cdi_final[,c("HospitalIDLabel", "date2", "HCFA_CDI_cases")], by=c("HospitalIDLabe
l", "date2"))

total_cdi$prop_ca <- total_cdi$CA_CDI_cases/(total_cdi$CA_CDI_cases+total_cdi$HCFA_CDI_cases)
total_cdi$total_cases <- total_cdi$CA_CDI_cases+total_cdi$HCFA_CDI_cases

range(total_cdi$date3)
```

```
## [1]  1 60
```

```
mean(total_cdi$CDiffTotalAdmissions)
```

```
## [1] 940.8445
```

```
total_cdi <- total_cdi %>% filter(total_cdi$total_cases>0)

# Check for missing data (CA, HCFA, or total):
summary(total_cdi)
```

© 2019 Turner NA et al. *JAMA Network Open*

```
##   HospitalID           Year             Month           CA_CDI_cases
##  Length:2055        Length:2055        Length:2055        Min.   : 0.000
##  Class :character   Class :character   Class :character   1st Qu.: 2.000
##  Mode  :character   Mode  :character   Mode  :character   Median : 4.000
##                                                           Mean   : 5.904
##                                                           3rd Qu.: 8.000
##                                                           Max.   :46.000
##
##    Molecular     HospitalIDLabel    CDiffTotalAdmissions CDiffPatientDays
##  Min.   :0.000   Length:2055        Min.   :  69.0       Min.   :  210
##  1st Qu.:1.000   Class :character   1st Qu.: 462.5       1st Qu.: 1884
##  Median :1.000   Mode  :character   Median : 816.0       Median : 3324
##  Mean   :0.763                      Mean   : 983.2       Mean   : 4268
##  3rd Qu.:1.000                      3rd Qu.:1272.0       3rd Qu.: 5374
##  Max.   :1.000                      Max.   :3934.0       Max.   :17533
##
##   CDiffLabIDHO       CDiffPCR          Beds           Urban
##  Min.   : 0.000   Min.   :0.000   Min.   : 48.0   Min.   :0.0000
##  1st Qu.: 1.000   1st Qu.:1.000   1st Qu.:142.0   1st Qu.:0.0000
##  Median : 2.000   Median :1.000   Median :214.0   Median :1.0000
##  Mean   : 2.691   Mean   :1.147   Mean   :261.1   Mean   :0.6998
##  3rd Qu.: 4.000   3rd Qu.:1.000   3rd Qu.:303.0   3rd Qu.:1.0000
##  Max.   :22.000   Max.   :2.000   Max.   :829.0   Max.   :1.0000
##                   NA's   :19
##    Academic           incidence      incidence_a          date
##  Length:2055        Min.   : 0.00   Min.   : 0.000   Min.   :2013
##  Class :character   1st Qu.: 6.80   1st Qu.: 2.800   1st Qu.:2014
##  Mode  :character   Median :12.70   Median : 5.290   Median :2016
##                     Mean   :15.35   Mean   : 6.516   Mean   :2016
##                     3rd Qu.:19.90   3rd Qu.: 8.440   3rd Qu.:2017
##                     Max.   :97.60   Max.   :41.150   Max.   :2018
##
##       date2          date3        HospitalID2     predicted
##  Apr 2016:  39   Min.   : 1.00   1001   :  60   Min.   :-1.423
##  Jun 2017:  39   1st Qu.:17.00   1003   :  60   1st Qu.: 0.711
##  Jul 2016:  38   Median :32.00   1004   :  60   Median : 1.388
##  Oct 2016:  38   Mean   :31.62   1011   :  60   Mean   : 1.375
##  Feb 2017:  38   3rd Qu.:46.00   1017   :  60   3rd Qu.: 2.102
##  Jan 2015:  37   Max.   :60.00   1019   :  60   Max.   : 3.566
##  (Other) :1826                   (Other):1695
##  predicted_rate    HCFA_CDI_cases      prop_ca        total_cases
##  Min.   : 0.5256   Min.   : 0.000   Min.   :0.0000   Min.   : 1.00
##  1st Qu.: 3.7213   1st Qu.: 1.000   1st Qu.:0.4091   1st Qu.: 3.00
##  Median : 5.4888   Median : 3.000   Median :0.5714   Median : 7.00
##  Mean   : 6.1834   Mean   : 4.312   Mean   :0.5654   Mean   :10.22
##  3rd Qu.: 7.1947   3rd Qu.: 6.000   3rd Qu.:0.7422   3rd Qu.:14.00
##  Max.   :38.5013   Max.   :33.000   Max.   :1.0000   Max.   :64.00
##
```

```
# Calculate proportions:
prop <- cbind(total_cdi$CA_CDI_cases, total_cdi$HCFA_CDI_cases)

# Mixed effects logistic regression using glmmTMB:

# Mixed effects modeling:
fit_logit <- glmmTMB(prop ~ date3 + (date3|HospitalID), data=total_cdi, family=binomial)
summary(fit_logit)
```

```
##  Family: binomial  ( logit )
## Formula:          prop ~ date3 + (date3 | HospitalID)
## Data: total_cdi
##
##      AIC      BIC   logLik deviance df.resid
##   7101.2   7129.3  -3545.6   7091.2     2050
##
## Random effects:
##
## Conditional model:
##  Groups     Name        Variance  Std.Dev. Corr
##  HospitalID (Intercept) 0.2631503 0.51298
##             date3       0.0001261 0.01123  -0.72
## Number of obs: 2055, groups:  HospitalID, 43
##
## Conditional model:
##             Estimate Std. Error z value Pr(>|z|)
## (Intercept) -0.035116   0.092655  -0.379    0.705
## date3        0.010291   0.002155   4.775  1.8e-06 ***
## ---
## Signif. codes:  0 '***' 0.001 '**' 0.01 '*' 0.05 '.' 0.1 ' ' 1
```

© 2019 Turner NA et al. *JAMA Network Open*

```
exp(confint(fit_logit))
```

```
##                                2.5 %    97.5 %  Estimate
## cond.(Intercept)           0.8051581 1.1577570 0.9654933
## cond.date3                 1.0060849 1.0146207 1.0103438
## cond.Std.Dev.(Intercept)   1.4514219 2.0266026 1.6702641
## cond.Std.Dev.date3         1.0078570 1.0162373 1.0112908
## cond.Cor.date3.(Intercept) 0.4229416 0.6932276 0.4861031
```

```
fit_logit_adj <- glmmTMB(prop ~ date3 + Urban + Academic + Molecular + (date3|HospitalID), data=total_cdi, family=binomial)
summary(fit_logit_adj)
```

```
##  Family: binomial  ( logit )
## Formula:
## prop ~ date3 + Urban + Academic + Molecular + (date3 | HospitalID)
## Data: total_cdi
##
##      AIC      BIC   logLik deviance df.resid
##   7104.2   7149.2  -3544.1   7088.2     2047
##
## Random effects:
##
## Conditional model:
##  Groups     Name        Variance Std.Dev. Corr
##  HospitalID (Intercept) 0.260561 0.51045
##             date3       0.000122 0.01105  -0.73
## Number of obs: 2055, groups:  HospitalID, 43
##
## Conditional model:
##                Estimate Std. Error z value Pr(>|z|)
## (Intercept)   -0.048157   0.146918  -0.328    0.743
## date3          0.009789   0.002189   4.472 7.76e-06 ***
## Urban          0.054809   0.132257   0.414    0.679
## AcademicMinor -0.166106   0.116848  -1.422    0.155
## Molecular      0.079355   0.091762   0.865    0.387
## ---
## Signif. codes:  0 '***' 0.001 '**' 0.01 '*' 0.05 '.' 0.1 ' ' 1
```

```
exp(confint(fit_logit_adj))
```

```
##                                2.5 %    97.5 %  Estimate
## cond.(Intercept)           0.7145440 1.2709895 0.9529837
## cond.date3                 1.0055132 1.0141786 1.0098366
## cond.Urban                 0.8151284 1.3689270 1.0563386
## cond.AcademicMinor         0.6735965 1.0649322 0.8469561
## cond.Molecular             0.9043898 1.2958984 1.0825883
## cond.Std.Dev.(Intercept)   1.4422534 2.0370706 1.6660433
## cond.Std.Dev.date3         1.0076835 1.0160713 1.0111082
## cond.Cor.date3.(Intercept) 0.4189838 0.6905756 0.4799174
```

```
# Code for plotting model predictions vs actual average proportions monthly:
tmpdat <- total_cdi[,c("prop_ca", "date3", "HospitalID", "Urban", "Academic")]
summary(tmpdat)
```

```
##     prop_ca           date3        HospitalID            Urban
##  Min.   :0.0000   Min.   : 1.00   Length:2055        Min.   :0.0000
##  1st Qu.:0.4091   1st Qu.:17.00   Class :character   1st Qu.:0.0000
##  Median :0.5714   Median :32.00   Mode  :character   Median :1.0000
##  Mean   :0.5654   Mean   :31.62                      Mean   :0.6998
##  3rd Qu.:0.7422   3rd Qu.:46.00                      3rd Qu.:1.0000
##  Max.   :1.0000   Max.   :60.00                      Max.   :1.0000
##    Academic
##  Length:2055
##  Class :character
##  Mode  :character
##
##
##
```

```
jvalues <- with(total_cdi, seq(from=min(date3), to=max(date3), length.out=60))

pred_prob <- lapply(jvalues, function(j){
  tmpdat$date3 <- j
  predict(fit_logit, newdata=tmpdat, type="response")
})

sapply(pred_prob[c(1:60)], mean)
```

```
##  [1] 0.4964934 0.4988068 0.5011254 0.5034487 0.5057765 0.5081085 0.5104443
##  [8] 0.5127836 0.5151261 0.5174714 0.5198192 0.5221692 0.5245211 0.5268745
## [15] 0.5292290 0.5315844 0.5339402 0.5362962 0.5386520 0.5410072 0.5433615
## [22] 0.5457146 0.5480661 0.5504157 0.5527630 0.5551077 0.5574495 0.5597880
## [29] 0.5621228 0.5644537 0.5667803 0.5691023 0.5714193 0.5737311 0.5760372
## [36] 0.5783375 0.5806316 0.5829191 0.5851998 0.5874733 0.5897394 0.5919978
## [43] 0.5942482 0.5964902 0.5987238 0.6009484 0.6031640 0.6053701 0.6075667
## [50] 0.6097533 0.6119299 0.6140961 0.6162517 0.6183965 0.6205303 0.6226528
## [57] 0.6247639 0.6268633 0.6289509 0.6310265
```

```
plotdata <- t(sapply(pred_prob, function(x){
  c(M=mean(x), quantile(x, c(0.25, 0.75)))
}))

plotdata <- as.data.frame(cbind(plotdata, jvalues))

colnames(plotdata) <- c("PredictedProbability", "LL", "UL", "date3")

ggplot(plotdata, aes(x=date3, y=PredictedProbability))+
  geom_line()+
  ylim(c(0,1))
```

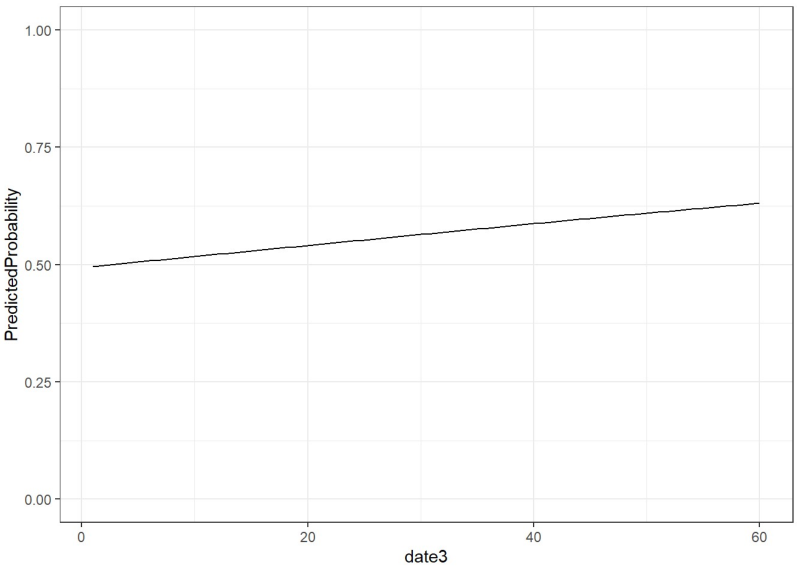


© 2019 Turner NA et al. *JAMA Network Open*

```r
# Merging in actual points:
attach(total_cdi)
mean_pct_ca <- aggregate(prop_ca, by=list(date3), FUN=mean, na.rm=TRUE)
pct_ca_sd <- aggregate(prop_ca, by=list(date3), FUN=sd, na.rm=TRUE)

colnames(mean_pct_ca) <- c("date3", "mean_pct_ca")
colnames(pct_ca_sd) <- c("date3", "sd")

pct_ca_agg2 <- left_join(mean_pct_ca, pct_ca_sd, by="date3")

pct_ca_agg2$ll2 <- pct_ca_agg2$mean_pct_ca-2*pct_ca_agg2$sd
pct_ca_agg2$ll2 <- ifelse(pct_ca_agg2$ll2<0, 0, pct_ca_agg2$ll2)
pct_ca_agg2$ul2 <- pct_ca_agg2$mean_pct_ca+2*pct_ca_agg2$sd

library(tidyverse)
complete_prop_data <- left_join(plotdata, pct_ca_agg2, by="date3")

#tiff('P:/DICON_OUTREACH/Research/Nick Turner ARLG Research/DICON CDI Basic Epi/figure_3b.tiff', units="in", width=10, heigh
t=6, res=300)

summary_prop_graph <- ggplot()+
  geom_point(data=complete_prop_data, aes(x=date3, y=100*mean_pct_ca))+
  geom_line(data=complete_prop_data, aes(x=date3, y=100*PredictedProbability), color="red")+
  geom_ribbon(data=complete_prop_data, aes(x=date3, ymin=LL, ymax=UL, alpha=0.1, fill="red"))+
  scale_x_continuous(name="Year", breaks=c(1,13,25,37,49), labels=c("2013", "2014", "2015", "2016", "2017"))+
  ylab("Proportion of CA-CDI Cases Relative to Total CDI Cases")+
  labs(NULL)+
  ylim(30,85)
summary_prop_graph
```

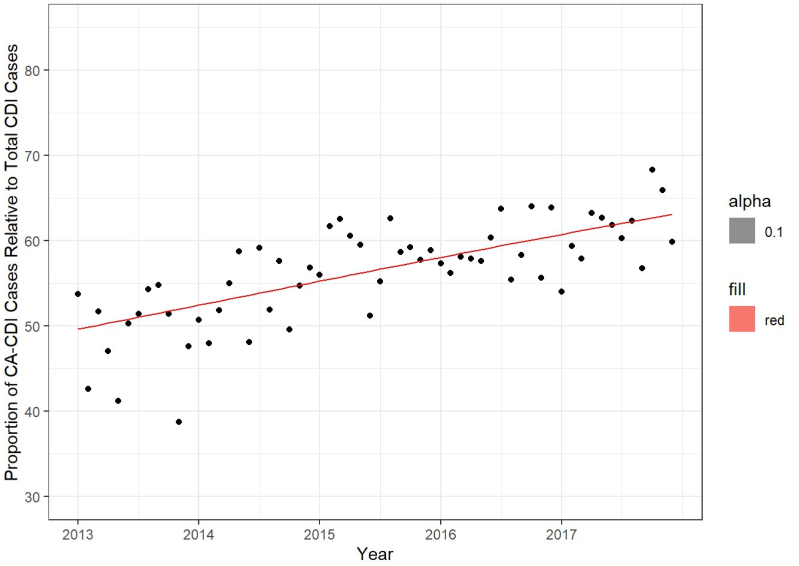


```r
#dev.off()

detach(total_cdi)

# Creation of combined plots:
library(gridExtra)
```

```
## Warning: package 'gridExtra' was built under R version 3.6.1
```

```
##
## Attaching package: 'gridExtra'
```

```
## The following object is masked from 'package:dplyr':
##
##     combine
```

© 2019 Turner NA et al. *JAMA Network Open*

```
ca_pred_plot3 <- ggplot(pred_ca_molec, aes(x=date3, y=predicted_rate))+
  geom_line()+
  geom_ribbon(aes(ymin=ll, ymax=ul), alpha=0.25)+
  scale_x_continuous(breaks=c(1,13,25,37,49), limits=c(0,60))+
  ylab("Modeled CA-CDI Rate (per 1,000 admissions)")+
  theme(axis.title.x = element_blank(), axis.text.x = element_blank())+
  labs(NULL)+
  ylim(0,30)
ca_pred_plot3
```

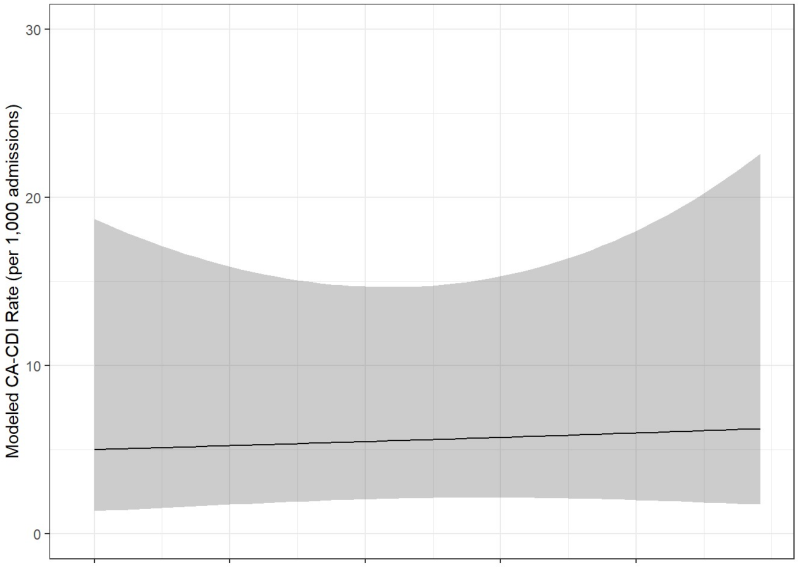


```
hcfa_pred_plot3 <- ggplot(pred_hcfa_molec, aes(x=date3, y=predicted_rate))+
  geom_line()+
  geom_ribbon(aes(ymin=ll, ymax=ul), alpha=0.25)+
  ylab("Modeled HCFA-CDI Rate (per 10,000 patient-days)")+
  theme(axis.title.x = element_blank(), axis.text.x = element_blank())+
  scale_x_continuous(breaks=c(1,13,25,37,49), limits=c(0,60))+
  labs(NULL)+
  ylim(0,30)
hcfa_pred_plot3
```

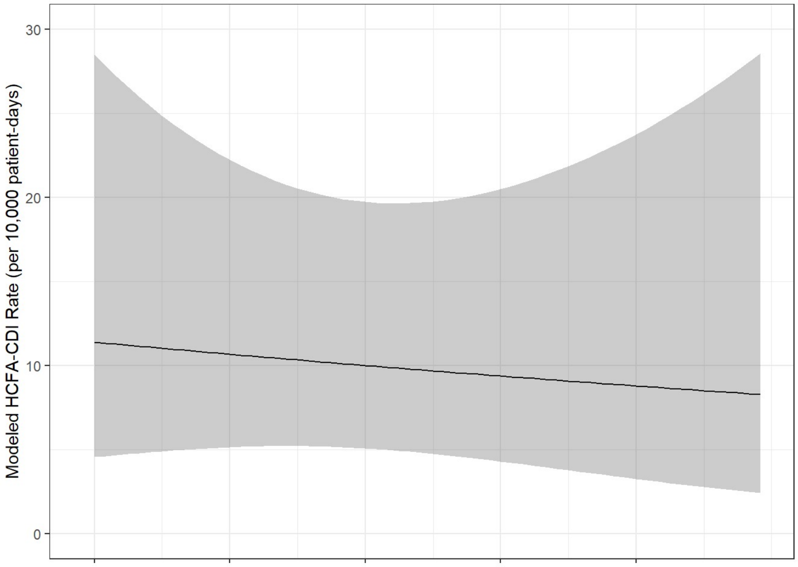


© 2019 Turner NA et al. *JAMA Network Open*

```
summary_prop_graph <- ggplot()+
  geom_point(data=complete_prop_data, aes(x=date3, y=100*mean_pct_ca))+
  geom_line(data=complete_prop_data, aes(x=date3, y=100*PredictedProbability), color="black")+
  geom_ribbon(data=complete_prop_data, aes(x=date3, ymin=100*LL, ymax=100*UL), alpha=0.1, color="grey", fill="grey")+
  scale_x_continuous(name="Year", breaks=c(1,13,25,37,49), limits=c(0,60), labels=c("2013", "2014", "2015", "2016", "201
7"))+
  scale_y_continuous(breaks=c(40,60,80), limits=c(30,85), labels=c(40,60,80))+
  ylab("Proportion of CA-CDI Cases Relative to Total (%)")+
  labs(NULL)
summary_prop_graph
```

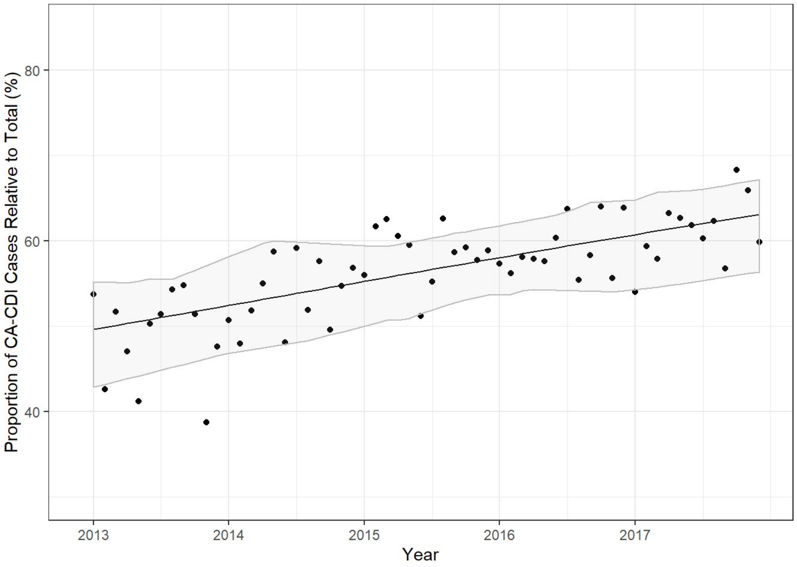


```
#pdf('P:/DICON_OUTREACH/Research/Nick Turner ARLG Research/DICON CDI Basic Epi/figure_3_combined.pdf', width=8, height=12)

#grid.arrange(ca_pred_plot3, hcfa_pred_plot3, summary_prop_graph, ncol=1, heights=c(1,1,1))

#dev.off()

#tiff('P:/DICON_OUTREACH/Research/Nick Turner ARLG Research/DICON CDI Basic Epi/figure_3_combined.tiff', units="in", width=
8, height=12, res=300)

#grid.arrange(ca_pred_plot3, hcfa_pred_plot3, summary_prop_graph, ncol=1, heights=c(1,1,1))

#dev.off()
```

© 2019 Turner NA et al. *JAMA Network Open*

**eFigure 1.** Total CDI Incidence by Year Among 43 DICON Hospitals[a]

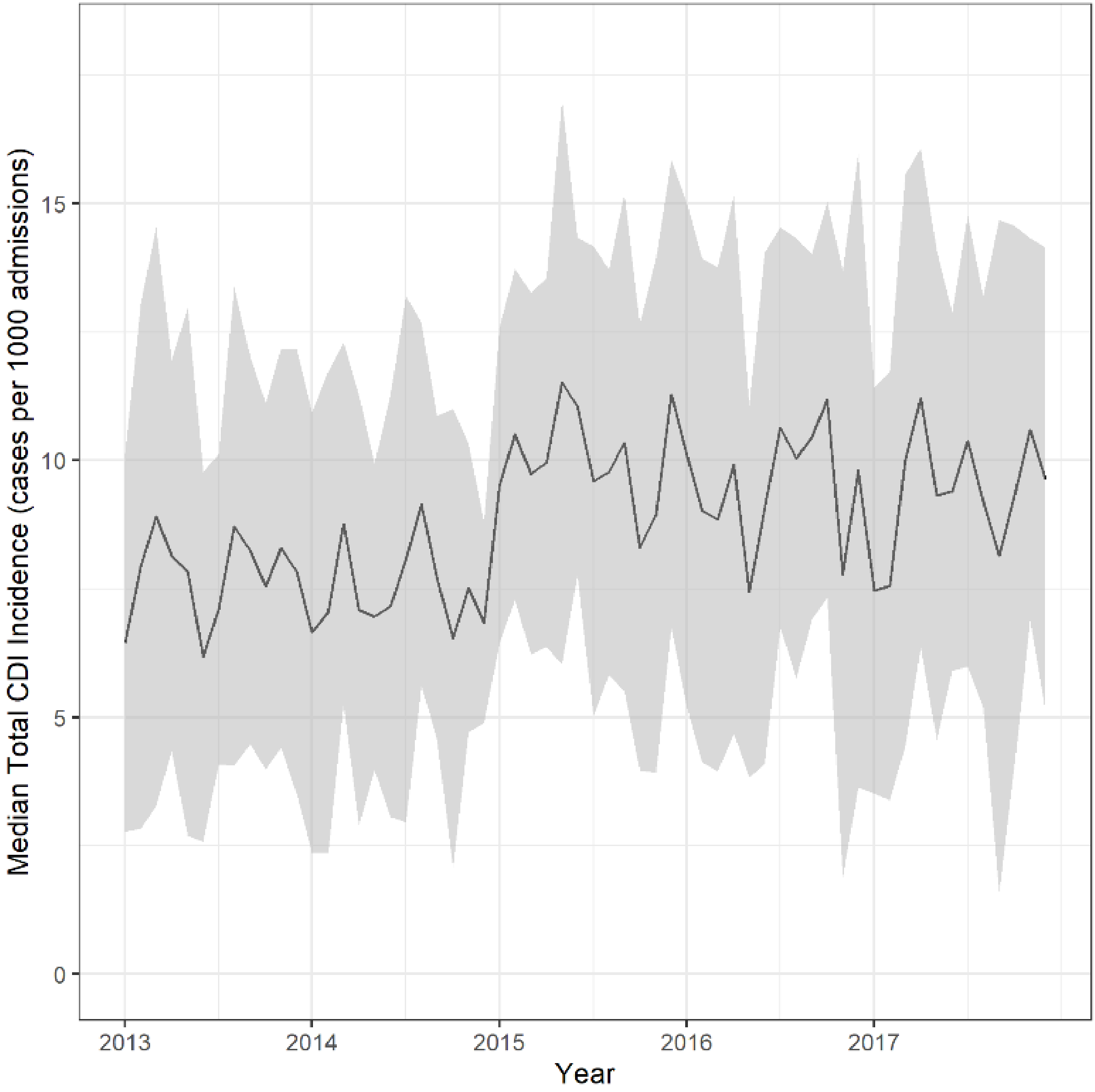


[a]Solid black lines represent unadjusted median; grey ribbon borders represent interquartile ranges.

© 2019 Turner NA et al. *JAMA Network Open*

**eFigure 2.** Variation in NAP1 Incidence Across Hospitals[a]

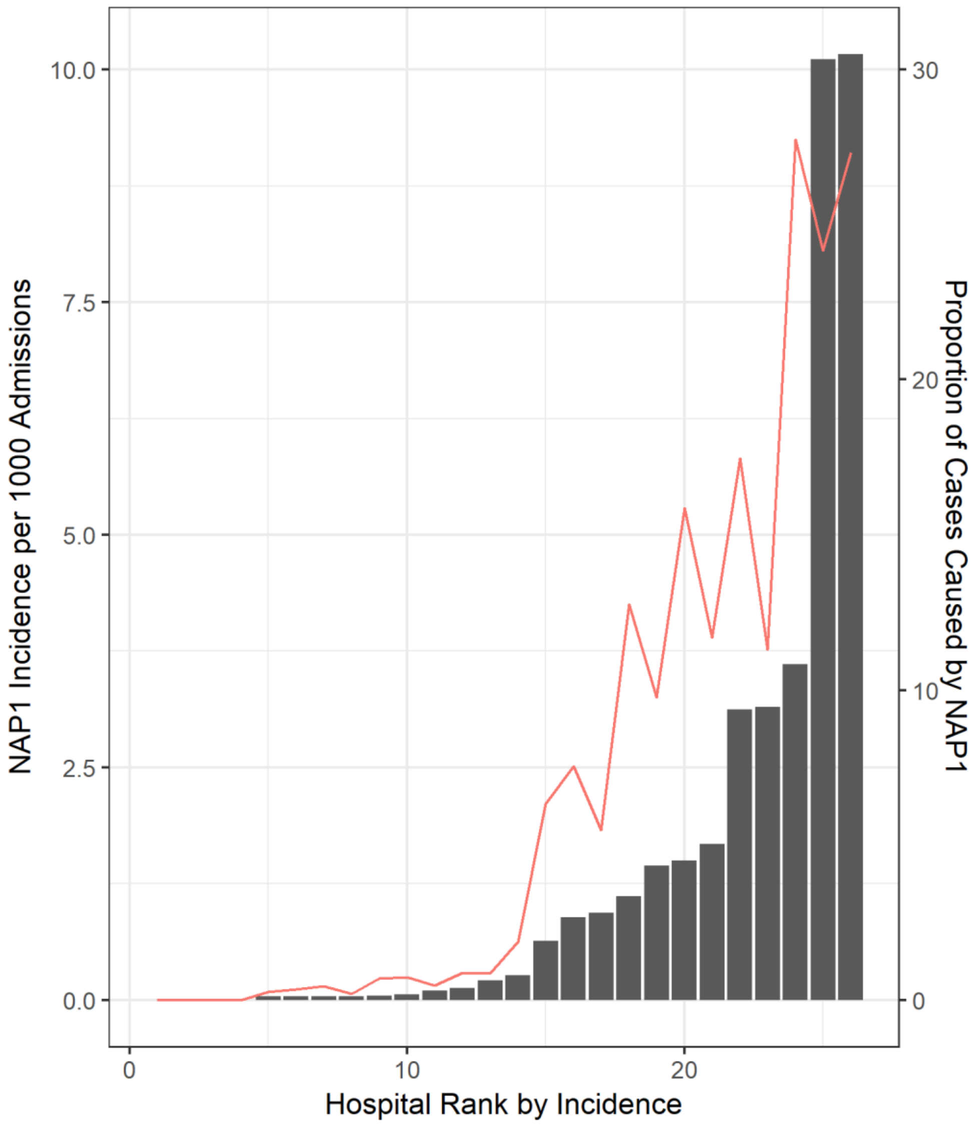


[a]Bar plot represents NAP1 incidence per 1000 admissions (leftward y-axis). Solid red line represents the proportion of total CDI cases caused by NAP1 strain (rightward y-axis).

© 2019 Turner NA et al. *JAMA Network Open*

**eFigure 3.** Trend in Time From Admission to CDI Testing Over the Study Period[a]

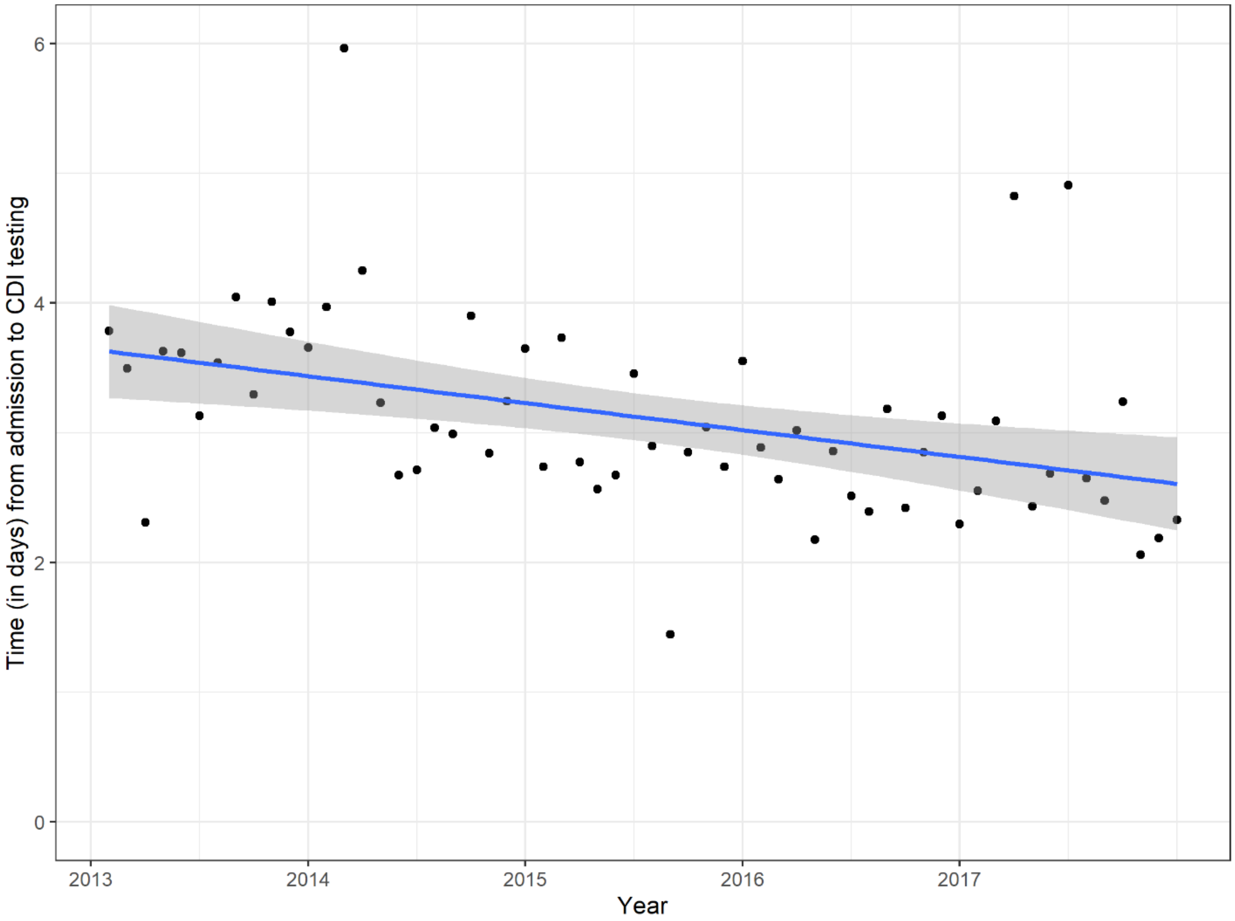


[a]Scatter plot points represent mean time (in days) from admission to CDI testing averaged across facilities. The blue line represents the linear regression model of time to testing over the study period, with slope representing a decrease in time to testing of 0.017 days/month (p=0.0017).

© 2019 Turner NA et al. *JAMA Network Open*

**eTable 1.** Demographic and Clinical Features of CA- and HCFA-CDI Cases

| **Clinical Characteristics** | **CA-CDI N=12,280 (%)[a]** | **HCFA-CDI N=8,974 (%)[a]** |
|---|---|---|
| **Age, median [Q1 – Q3][b]** | 67 [53-79] | 70 [58 – 80] |
| **Gender, female** | 7,515 (61.2) | 5,163 (57.5) |
| Not reported/missing | 3 (0.02) | 5 (0.06) |
| **Race** | | |
| African-American | 1,899 (15.5) | 2,083 (23.2) |
| Asian | 21 (0.2) | 25 (0.3) |
| Hispanic | 50 (0.4) | 57 (0.6) |
| Caucasian | 5,353 (43.6) | 4,128 (46.0) |
| Other | 189 (1.5) | 170 (1.9) |
| Not reported/missing | 4,768 (38.8) | 2,511 (28.0) |
| **Admitted from:** | | |
| Home | 7,497 (61.1) | 5,173 (57.6) |
| Other hospital | 148 (1.2) | 298 (3.3) |
| Skilled nursing facility | 1,138 (9.3) | 1,635 (18.2) |
| Extended care facility | 216 (1.8) | 311 (3.5) |
| Home health | 94 (0.8) | 134 (1.5) |
| Other | 90 (0.7) | 113 (1.3) |
| Not reported/missing | 3,097 (25.2) | 1,310 (14.6) |
| **NAP1 positive[c]** | 461 (3.8) | 565 (6.3) |
| NAP1 negative | 2,058 (16.8) | 1,914 (21.3) |
| Not reported/missing | 9,761 (79.5) | 6,495 (72.4) |

[a]Due to rounding, percentages may not sum to exactly 100%.

[b] Q1 = 1st quartile, Q3 = 3rd quartile

[c]Reporting was voluntary and only performed by 26 of the 43 total hospitals. As such, there is some risk of selection bias in NAP1 reporting rates.

© 2019 Turner NA et al. *JAMA Network Open*

**eTable 2.** Results of Modeling CA- and HCFA-CDI Incidence Rates Over Time, Stratified by Whether Test Method Changed Over Time

| Model | Subset | Fixed Effects | Incidence Rate Ratio (95% CI) | p-value |
|---|---|---|---|---|
| **CA-CDI** | No change in testing | Time (months) | 1.001 (0.995-1.007) | 0.79 |
| | Testing updated | Time (months) | 1.021 (1.013-1.030) | <0.001 |
| **HCFA-CDI** | No change in testing | Time (months) | 0.993 (0.986-0.999) | 0.02 |
| | Testing updated | Time (months) | 1.010 (1.002-1.017) | 0.01 |

© 2019 Turner NA et al. *JAMA Network Open*

**eTable 3.** Results of Sensitivity Analysis[a]

| Model | Fixed Effects | Incidence Rate Ratio (95% CI) | p-value |
|---|---|---|---|
| CA-CDI | Time (months) | 1.005 (0.998-1.011) | 0.17 |
| | Setting (urban relative to rural) | 0.826 (0.577-1.185) | 0.30 |
| | Type (academic relative to community) | 1.091 (0.780-1.525) | 0.61 |
| | Test type (NAAT relative to antigen) | 1.967 (1.623-2.384) | <0.01 |
| HCFA-CDI | Time (months) | 0.997 (0.992-1.002) | 0.19 |
| | Setting (urban relative to rural) | 0.760 (0.573-1.009) | 0.06 |
| | Type (academic relative to community) | 0.969 (0.744-1.261) | 0.81 |
| | Test type (NAAT relative to antigen) | 2.022 (1.700-2.406) | <0.01 |

[a]A post-hoc sensitivity analysis was conducted to address the potential for misclassification of CA- versus HCFA-CDI cases by the LabID definition. For this analysis, more stringent definitions for HCFA-CDI were applied - including all patients admitted from a skilled nursing facility, extended care facility, or other hospital.

© 2019 Turner NA et al. *JAMA Network Open*
